# Structural basis for substrate recognition and inhibition of human glucose-6-phosphate transporter SLC37A4

**DOI:** 10.1371/journal.pbio.3003833

**Published:** 2026-07-28

**Authors:** Hui Li, Xiaomin Peng, Yuwen Huang, Wencheng Wu, Nan Li, Ziqi Cheng, Xuepeng Wei

**Affiliations:** 1 GMU-GIBH Joint School of Life Sciences, Guangdong Provincial Key Laboratory of Protein Modification and Disease, The Guangdong-Hong Kong-Macao Joint Laboratory for Cell Fate Regulation and Diseases, Guangzhou Medical University, Guangzhou, Guangdong, China; 2 Guangzhou National Laboratory, Guangzhou, Guangdong, China; 3 University of Chinese Academy of Sciences, Beijing, China; 4 Zhongshan School of Medicine, Sun Yat-sen University, Guangzhou, Guangdong, China; University of Zurich, SWITZERLAND

## Abstract

Glucose 6 phosphate (G6P) homeostasis is essential for maintaining blood glucose levels and coordinating anabolic and catabolic pathways. A key step in this process is the delivery of G6P into the endoplasmic reticulum (ER), where it is hydrolyzed by glucose 6 phosphatase to glucose and inorganic phosphate (Pi). This transport step is carried out by the ER carrier SLC37A4 (also known as the G6P transporter, G6PT), which imports G6P into the ER lumen while exporting Pi to the cytosol, and loss-of-function mutations in SLC37A4 cause glycogen storage disease type Ib. Despite its central role in G6P homeostasis, how SLC37A4 recognizes G6P and couples its transport to Pi antiport has remained unclear. Here we report cryo-electron microscopy structures of human SLC37A4 in three states: the apo form at 2.8 Å resolution, a G6P-bound state at 3.2 Å resolution and a chlorogenic acid (CHA) bound state at 3.3 Å resolution. SLC37A4 adopts the canonical Major Facilitator Superfamily fold and harbors a central, positively charged cavity that accommodates anionic substrates. In the G6P-bound structure, SLC37A4 adopts an outward-open conformation facing the ER lumen, in which G6P binds to the electropositive pocket. In the CHA-bound structure, SLC37A4 adopts an inward-facing conformation, with CHA bound at a cytosolic site that locks the transporter in an arrested state and prevents the conformational transitions required for G6P/Pi exchange. Combined with thermostability and transport-based analyses of G6P binding and disease variants, these structures support a rocker switch mechanism in which electrostatic neutralization of the central positively charged cavity by anionic substrate drives the conformational changes underlying G6P/Pi exchange. Together, these findings define the structural basis of G6P/Pi exchange by SLC37A4, provide a molecular rationale for pathogenic mutations in glycogen storage disease type Ib, and provide a framework for targeting SLC37A4 to modulate G6P homeostasis.

## Abstract 2

The human glucose-6-phosphate transporter (SLC37A4) mediates the translocation of G6P into the ER, but the structural basis of G6P recognition is unclear. This study solves high-resolution cryo-EM structures of SLC37A4 in three conformational states and provides a structural framework for substrate recognition and inhibition.

## Introduction

Glucose-6-phosphate (G6P) serves as a critical metabolic hub at the intersection of several major pathways, seamlessly linking glycolysis, the pentose phosphate pathway, and glycogen metabolism to maintain cellular energy balance [1–3]. Following cellular uptake, free glucose is immediately phosphorylated to G6P by the rate-limiting enzymes hexokinase and glucokinase, a phosphorylation event that effectively traps glucose within the cell [4–6]. While cytosolic metabolism allows G6P to direct carbon flux toward immediate energy production, redox balance, or biosynthesis, G6P homeostasis crucially depends on its transport into the endoplasmic reticulum (ER) lumen to support systemic glucose release. This translocation is mediated by SLC37A4 (also known as the glucose-6-phosphate transporter, or G6PT), which functions as an obligate antiporter, moving cytosolic G6P into the ER while simultaneously exporting inorganic phosphate (Pi) back to the cytosol [7,8]. Once inside the ER, G6P is hydrolyzed by glucose-6-phosphatase into glucose and Pi, which are then returned to the cytosol to enable glucose efflux into the circulation.

The physiological necessity of SLC37A4/G6PT is illustrated by the consequences of its failure, particularly in the context of glycogen storage disease type Ib (GSD Ib). Dysfunction of SLC37A4 disrupts the delicate transport process, leading directly to this rare, autosomal recessive disorder [9–12]. Despite the critical role of SLC37A4 in human physiology and disease, the molecular mechanisms by which it selectively recognizes G6P, coordinates the precise exchange of G6P and Pi, and undergoes the necessary conformational transitions for transport have historically remained poorly understood.

In this study, we report high-resolution cryo-electron microscopy (cryo-EM) structures of human SLC37A4 in three distinct functional states: the apo state, the G6P-bound state, and an inhibitor-bound state complexed with chlorogenic acid (CHA). By integrating these structural insights with thermostability-shift and transport assays characterizing G6P binding and function, we elucidate the molecular mechanism of substrate recognition and conformational cycle underlying G6P transport, demonstrating how the transporter navigates between inward- and outward-facing conformations to facilitate the exchange of G6P and Pi. The CHA-bound structure further reveals a cytosolic inhibitory site and defines how CHA prevents the conformational transitions required for transport. Ultimately, this work provides a comprehensive structural framework for understanding SLC37A4 function, offers a molecular rationale for pathogenic mutations in GSD Ib, and lays the foundation for therapeutic development targeting SLC37A4.

## Results

### Overall structure of SLC37A4

**Fig 1:**
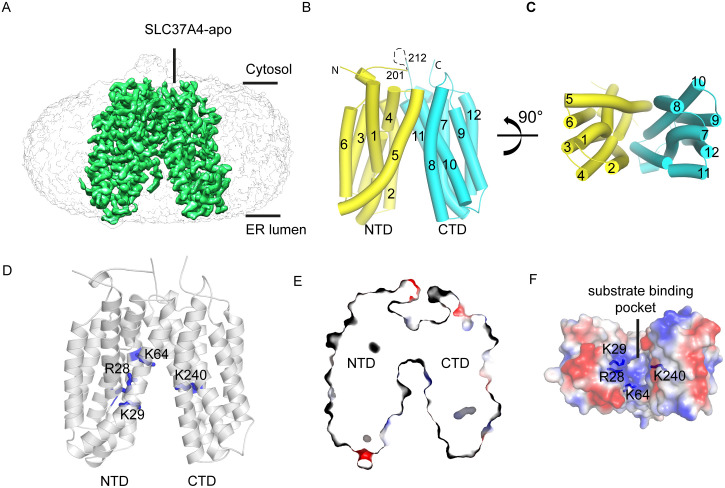
Overall structure of human SLC37A4 in the apo state. **(A)** Cryo-EM density map of SLC37A4-apo viewed along the membrane plane. The protein density is shown in green and the detergent micelle shown in transparent gray. **(B)** Cartoon representation of SLC37A4 structure in the same membrane plane orientation as in **a.** N-terminal domain NTD is shown in yellow and C-terminal domain CTD is shown in cyan, individual transmembrane helices are numbered. The dashed line indicates an unresolved cytosolic loop between residues 201 and 212 that connects TM6 and TM7, linking the NTD and CTD. **(C)** View of SLC37A4 from the ER-lumen side along membrane normal, obtained by a 90° rotation of the model in b, showing the arrangement of the NTD and CTD. **(D)** Overall structure of SLC37A4 in the orientation in a, highlighting 4 positively charged residues in the putative substrate-binding pocket. **(E)** Cutaway view in the orientation of a, showing a slice through the putative substrate binding pocket. **(F)** Electrostatic surface representation of SLC37A4 viewed from the same orientation in c, ER-lumen side along the membrane normal, showing the putative substrate-binding pocket and the location of R28, K29, K64 and K240.

To elucidate the structural basis of G6P transport, we expressed SLC37A4 in HEK293 cells, purified the protein in the detergent lauryl maltose neopentyl glycol (LMNG) and determined its structure using single particle cryo-EM (S1 Fig). Although 2D classification revealed the presence of both monomeric and dimeric species, iterative rounds of heterogeneous refinement enabled isolation of the monomeric particles and yielded a reconstruction of the apo state at 2.8 Å resolution (S2 Fig and Table 1). The SLC37A4 structure exhibits the canonical fold of the Major Facilitator Superfamily (MFS), comprising 12 transmembrane helices (TMs). These helices are organized into two distinct domains, an N-terminal domain (NTD; TM1–6) and a C-terminal domain (CTD; TM7–12), which are connected by a highly flexible cytosolic loop. Because of this flexibility, the residues 202–211 were not resolved in the cryo-EM map and were omitted from the final model. Together, the NTD and CTD display the characteristic pseudo 2-fold symmetry of the MFS family (Fig 1A–1C).

**Table 1:** Cryo-EM data collection, refinement, and validation statistics.

	SLC37A4-apo (EMDB-67847) (PDB 21NQ)	SLC37A4-G6P (EMDB-67869) (PDB 21OR)	SLC37A4-CHA (EMDB-67846) (PDB 21NP)
**Data collection and processing**			
Magnification	215,000	215,000	215,000
Voltage (kV)	300	300	300
Electron exposure (e−/Å^2^)	50	50	50
Defocus range (μm)	0.8-1.6	0.8-1.6	0.8-1.6
Pixel size (Å)	0.578	0.578	0.578
Symmetry imposed	C1	C1	C1
Initial particle images (no.)	5,769,272	5,093,164	4,959,911
Final particle images (no.)	115,232	149,880	82,953
Map resolution (Å)	2.8	3.2	3.3
FSC threshold	0.143	0.143	0.143
Map resolution range (Å)	2.7-3.5	2.8-4.0	2.8-4.0
**Refinement**			
Initial model used (PDB code)	Alphafold	Alphafold	Alphafold
Model resolution (Å)	2.9	3.3	3.5
FSC threshold	0.5	0.5	0.5
Model composition			
Non-hydrogen atoms	3,159	3,175	3,244
Protein residues	414	414	422
Ligands	0	1	1
*B* factors (Å^2^)			
Protein	128.08	119.11	105.31
Ligand	–	200.24	107.84
R.m.s. deviations			
Bond lengths (Å)	0.003	0.003	0.004
Bond angles (°)	0.671	0.754	0.669
Validation			
MolProbity score	1.48	1.84	1.89
Clashscore	6.33	14.65	8.96
Poor rotamers (%)	1.5	0.6	0.9
Ramachandran plot			
Favored (%)	98.05	97.07	94.05
Allowed (%)	1.95	2.93	5.95
Disallowed (%)	0	0	0

In the apo state, SLC37A4 adopts a wide outward-open conformation facing the ER lumen. This conformation likely represents the state for either G6P release into the lumen or subsequent loading of Pi, corresponding to the stage of the transport cycle prior to resetting to the cytosol-facing state (Fig 1B and 1C). Electrostatic potential surface analysis reveals a positively charged cavity in SLC37A4, primarily generated by four key basic residues: R28 and K29 on TM1, K64 on TM2, and K240 on TM7(Fig 1C). Given that both G6P and Pi are negatively charged, we speculate that this positively charged pocket is responsible for substrate binding (Fig 1D–1F).

### Structure of SLC37A4 bound to G6P substrate

**Fig 2:**
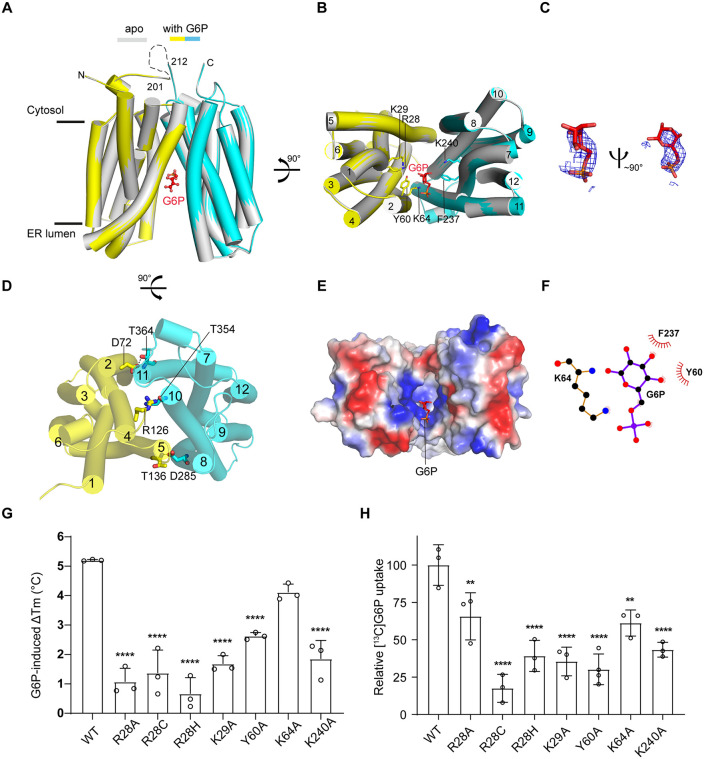
Structural basis of G6P recognition by SLC37A4. **(A)** Superposition of the SLC37A4 apo state (gray) and the G6P-bound state (NTD in yellow, CTD in cyan). The structures are shown as cylindrical cartoons viewed along the membrane plane. The bound G6P molecule is shown as red sticks in the central cavity. The dashed line indicates an unresolved cytosolic loop between residues 201 and 212 that connects TM6 and TM7, linking the NTD and CTD. **(B)** View of the superposed apo and G6P-bound structures from the ER-lumen side along the membrane normal, highlighting G6P and nearby residues R28, K29, Y60, K64, F237 and K240. **(C)** Cryo-EM density of the bound G6P molecule, with a contour level of 3σ. **(D)** View of the interface between the N- and C-terminal domains from the cytosolic side along the membrane normal, showing gating residues at the domain interface, including D72, R126, T136, D285, T354 and T364. **(E)** Electrostatic surface representation of SLC37A4 viewed from the same orientation in **b. (F)** Two-dimensional schematic of the G6P-binding sites, depicting hydrogen-bond interactions between G6P and K64, as well as hydrophobic contacts with F237 and Y60. **(G)** GFP-based thermostability assay showing G6P-induced changes in melting temperature (ΔTm) for wild type (WT) SLC37A4 and G6P-binding–site mutants (R28A, R28C, R28H, K29A, Y60A, K64A, K240A). Bars represent mean ± s.d. of measurements performed at least in triplicate. The underlying data for this figure can be found in S1 Data. **(H)** Relative [^13^C_6_]G6P uptake for WT and mutant SLC37A4. Bars represent mean ± s.d. of measurements performed at least in triplicate. The underlying data for this figure can be found in S1 Data.

To elucidate the molecular basis of substrate recognition, we determined the structure of SLC37A4 in the presence of G6P at 3.2 Å resolution (S3 Fig and Table 1). Superposition of the apo and G6P-bound structures shows that the overall fold is nearly identical, with a root-mean-square deviation (RMSD) of 0.29 Å between Cα atoms, indicating that substrate binding does not induce a global conformational change (Fig 2A and 2B). Both structures were captured in an outward-facing conformation, which appears to be stabilized by extensive NTD-CTD interactions on the cytosolic side (Fig 2D). Hydrogen bonds and salt bridges are formed between D72 on TM2 and T364 on TM11, R126 on TM4 and T354 on TM10, as well as T136 on TM5 and D285 on TM8. These contacts likely stabilize the NTD and CTD in an arrangement that keeps the transporter open to the ER lumen (Fig 2D).

Closer inspection of the positively charged cavity revealed additional density not present in the apo map (Figs 2C and S4). This density is located within the central cavity below the NTD-CTD interface (Fig 2A and 2B). This density lies within about 3 Å of K64 on TM2, and is also closely surrounded by R28 and K29 on TM1, Y60 on TM2 and F237 and K240 on TM7 (Fig 2B). Based on the size, shape, and the local chemical environment, we tentatively assigned this feature as the bound G6P substrate. The phosphate group of G6P forms hydrogen bonds with the side chain of K64, whereas the sugar moiety forms hydrophobic interactions with bulky residues Y60 and F237 (Fig 2B and 2F). Collectively, these residues define a positively charged, polar-aromatic pocket (Fig 2E) that accommodates the phosphate and glucose moieties of G6P. We generated alanine mutants and assessed their thermostability to validate the roles of these potential substrate-binding residues. The observed reduction in thermal shift upon substrate addition confirmed that these residues are indeed responsible for substrate binding (Figs 2G and S5). Furthermore, despite comparable protein expression levels across all mutants (S6 Fig), these mutations significantly impaired G6P transport activity, as demonstrated by a relative [^13^C_6_]G6P uptake assay (Fig 2H). Notably, the naturally occurring disease-related mutations R28C and R28H also exhibited severe defects in both substrate binding and transport (Fig 2G and 2H).

### Structure of SLC37A4 bound to inhibitor CHA

Previous studies identified CHA and its analog as potent inhibitors of SLC37A4 [13–16]. Hypothesizing that inhibitor binding might trap the transporter in a distinct conformational state, allowing us to capture an alternative stage of the transport cycle and elucidate the structural basis of G6P transport, we sought to determine the structure of the CHA-bound complex. To address the heterogeneity observed in the LMNG detergent-solubilized protein, specifically the co-existence of monomeric and dimeric species, we reconstituted purified SLC37A4 into lipid nanodiscs. This approach yielded a highly monodisperse sample, which was then incubated with the inhibitor (S1 Fig). Subsequent 2D classification and heterogeneous refinement confirmed that the SLC37A4-CHA complex exists as a homogeneous monomer. Consequently, we were able to determine the structure of the inhibitor-bound complex to a resolution of 3.3 Å (S7 Fig and Table 1).

**Fig 3:**
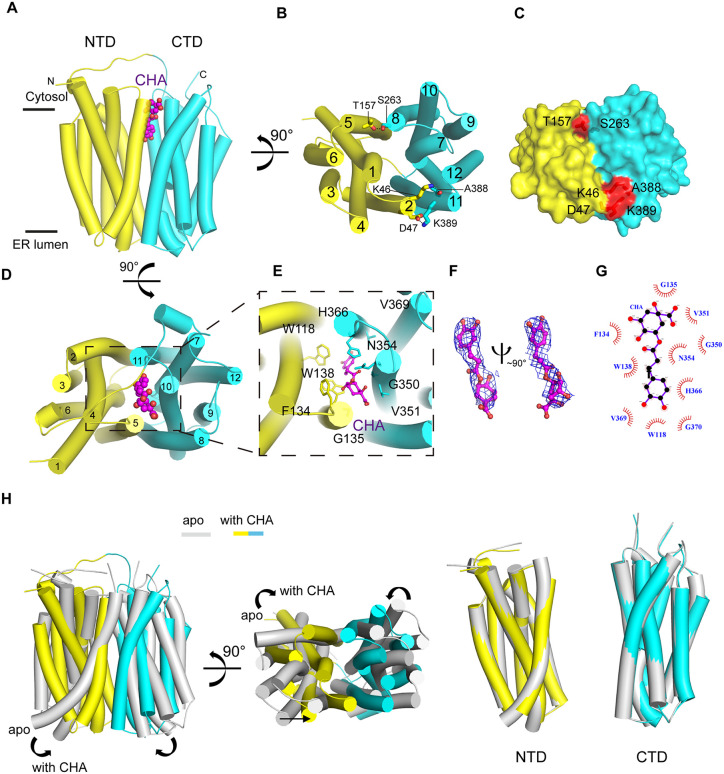
Structural basis of inhibition of SLC37A4 by CHA. **(A)** Overall structure of CHA-bound SLC37A4 shown as a cylindrical cartoon in the cytosol facing conformation. The N-terminal domain NTD is in yellow and the C-terminal domain CTD is in cyan. The cytosolic loop connecting the NTD and CTD is resolved in this structure. The bound inhibitor CHA is shown in magenta. **(B)** View of the NTD–CTD interface from the ER-lumen side along the membrane normal, highlighting polar and charged residues at the interdomain interface, including K46, D47, T157 in the NTD and S263, A388 and K389 in the CTD. **(C)** Surface representation of SLC37A4 in the same orientation as in b, showing the interdomain interface patch formed by T157, K46 and D47 in the NTD and S263, A388 and K389 in the CTD. **(D)** View of SLC37A4 from the cytosolic side along the membrane normal, showing the CHA was trapped at the interface of NTD and CTD. **(E)** Close-up view of the CHA-binding pocket, highlighting residues that form hydrophobic interactions with CHA, including W118, F134, W138 and G135 from the NTD and N354, G350, V351, H366 and V369 from the CTD. **(F)** Cryo-EM density of the bound CHA molecule, with a contour level of 8σ. **(G)** Two-dimensional schematic of the CHA-binding site, illustrating hydrophobic contacts and van der Waals interactions between CHA and surrounding residues G135, F134, W138, W118, N354, G350, V351, H366, V369 and G370. **(H)** Structural comparison of the apo (gray) and CHA-bound (yellow/cyan) SLC37A4 structures, viewed from the membrane plane (left), from the cytosol (middle), and separated into NTD and CTD (right), illustrating that CHA-binding induces a rigid-body rotation between the two domains without major intra-domain rearrangement.

In contrast to the apo and G6P-bound structures, the CHA-bound structure adopts an inward-facing conformation. Notably, the cytosolic loop between TM6 and TM7, which was highly flexible and unresolved in the apo state, is well-resolved in this inhibitor-bound structure (Fig 3A). This state is defined by the tight closure of the gate on the ER lumen side, which prevents access from the luminal compartment (Fig 3A–3C). This closure is maintained by specific interactions between the NTD and CTD. Key stabilizing features include a salt bridge between D47 on TM2 and K389 on TM11 (Fig 3B and 3C). In addition, the bottom of the transporter is sealed by hydrogen bonds between K46 on TM2 and A388 on TM11, and between T157 on TM5 and S263 on TM8. Surface representation confirms that these residues create a tightly sealed interface, effectively locking the ER luminal gate.

In this inward-facing conformation, a clear ligand-like density is observed on the cytosolic side at the NTD–CTD interface, which can be well fitted with a CHA molecule. CHA occupies a cavity near the cytosolic entrance at this interface, wedged between TM4–TM5 of the NTD and TM8–TM10–TM11 of the CTD (Fig 3D–3G). The inhibitor is surrounded by residues that engage in predominantly hydrophobic, van der Waals, and aromatic interactions, including W118, F134, W138, and G135 from the NTD, and V351, N354, H366, V369, and G370 from the CTD. Together, these residues form an extensive hydrophobic and aromatic interaction network with CHA, creating a non-polar pocket that matches the shape of CHA and explaining its high affinity and ability to stabilize the inward-facing conformation.

Compared with the apo structure, which is in an outward-open conformation facing the ER lumen, the CHA-bound structure is cytosol-facing (Fig 3H, left). Overlay of the CHA-bound and apo structures shows that the NTD and CTD themselves undergo no significant conformational changes within each domain (Fig 3H, right). Instead, the two domains move largely as rigid bodies relative to one another during the transition between ER lumen-facing and cytosol-facing conformations (Fig 3H). This is consistent with the classic rocker switch mechanism of MFS transporters [17–19].

### Conformational comparison reveals a putative G6P translocation tunnel in SLC37A4

**Fig 4:**
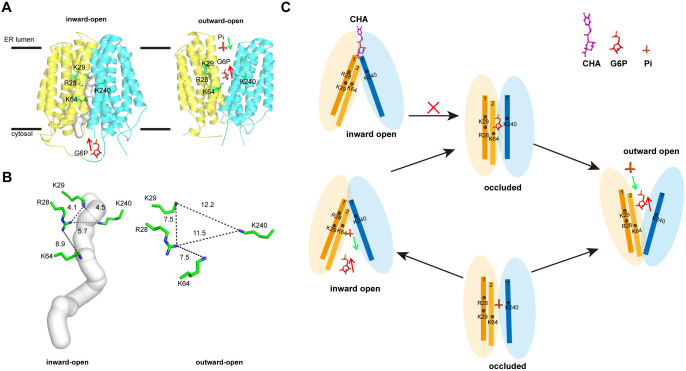
Conformational transitions and mechanistic model of substrate transport and inhibition. **(A)** Structural models of SLC37A4 in inward-open (left) and outward-open (right) conformations viewed in the membrane plane. The N-terminal domain (NTD) and C-terminal domain (CTD) are shown as yellow and cyan cartoons, respectively; horizontal black lines indicate the approximate membrane boundaries. The cluster of basic residues in the NTD (K29, R28 and K64) and K240 in the CTD are shown as green sticks. The putative translocation pathway is depicted as a transparent surface. In the inward-open state, G6P (red sticks) binds from the cytosolic side to the NTD basic cluster, while K240 in the CTD faces the cytosolic side. In the outward-open state, reorientation of the NTD and CTD exposes the binding site to the lumen, allowing G6P release and Pi binding, as indicated by arrows. **(B)** Close-up views of the basic residues in the inward-open (left) and outward-open (right) conformations. Distances (Å) between side-chain nitrogen atoms are indicated by dashed lines, highlighting the compact arrangement of K29, R28 and K64 in the inward-open state and their rearrangement relative to K240 upon transition to the outward-open state. **(C)** The schematic illustrates the conformational transitions between the inward-open, occluded, and outward-open states. The inhibition scenario (top) shows the binding of CHA to the inward-open state, which sterically hinders the transition to the occluded state (indicated by the red cross), thereby locking the transporter. In contrast, the functional antiport cycle (bottom) depicts the exchange of substrates: G6P binds to the inward-open state, facilitating the transition to the occluded state. Subsequently, the transporter shifts to the outward-open conformation to release G6P and accommodate the binding of inorganic phosphate (Pi) for transport in the reverse direction. Key residues (R28, K29, K64 on TM 1/2 and K240 on TM 7) involved in substrate/inhibitor recognition are highlighted.

Although we lack an experimental structure of inward-facing SLC37A4 bound to G6P, the competitive inhibitor CHA captures the transporter in a distinct inward-facing state. We reasoned that the structure obtained by manually removing CHA serves as a plausible candidate representing the conformation primed for G6P loading from the cytosol. Consequently, we utilized this *in silico* CHA-removed model to characterize the potential G6P transport pathway using the MOLEonline server [20]. Notably, with the ER luminal gate sealed in the inward-open state, K64 sits at the cytosolic entrance of the cavity rather than at its base, whereas R28, K29 and K240 occupy the base of the cavity. These three residues lie within 5–6 Å of one another and form a triangular, positively charged trap for G6P engagement (Fig 4A and 4B). We propose that G6P binding to this deep pocket initiates the transport cycle by neutralizing the local positive charge. Engagement of the phosphate by R28 and K29 likely stabilizes tighter NTD–CTD contacts on the cytosolic side, favoring the formation of an occluded intermediate in which both gates are transiently closed. This ligand-stabilized closure is then coupled to the canonical MFS rocker-switch motion, driving a rigid-body rearrangement of the two helical bundles that seals the cytosolic gate and remodels the luminal side to generate an outward-facing state.

In the outward-facing conformation represented by the apo and G6P-bound structures, the geometry of the basic cluster shifts and R28, K29 and K240 move apart, resulting in decreased affinity for G6P. The weakened affinity promotes G6P release into the ER lumen for subsequent hydrolysis by glucose-6-phosphatase. Meanwhile, the electropositive cavity is poised to bind Pi in the ER lumen, likely engaging the same basic residues, which in turn stabilizes the return transition back toward an inward-open state. Pi release to the cytosol would complete the exchange and reset the pocket for another round of G6P/Pi exchange.

## Discussion

In this study, we determined high-resolution cryo-EM structures of human SLC37A4 in apo, G6P-bound and CHA-bound states. Based on these structural snapshots together with our thermostability data, we propose a stepwise model for the G6P/Pi exchange mechanism (Fig 4C). The cycle likely begins from an inward-open conformation in which the basic cluster is arranged to favor high-affinity G6P binding from the cytosol. Substrate engagement is expected to promote compaction of the cytosolic cavity and formation of an occluded intermediate, in which both gates are transiently closed. Transition from this occluded state to an outward-facing conformation then proceeds through the canonical rocker-switch motion characteristic of Major Facilitator Superfamily transporters, reorienting the same electropositive cavity toward the ER lumen. In the outward-facing state, subtle rearrangements of the basic residues would lower G6P affinity, promoting its release for hydrolysis by glucose-6-phosphatase, while leaving the cavity primed for luminal Pi binding. Engagement of Pi would favor the reverse transition back toward the inward-open state, completing the exchange and resetting the transporter for another cycle.

Our inhibitor-bound structure illustrates how these conformational changes can be selectively interrupted. CHA stabilizes an inward-facing state that remains competent for cytosolic access yet is conformationally trapped and unable to progress toward the occluded or outward-facing configurations required for transport (Fig 4C). Rather than simply competing with G6P at the same coordination site, CHA engages an adjacent hydrophobic–aromatic pocket at the cytosolic entrance and locks inter-domain contacts. This mode of inhibition reveals a druggable site that could be exploited to modulate G6P flux.

To date, 54 pathogenic mutations have been reported in *SLC37A4* [9]. By leveraging the structural roles identified here, these known pathogenic missense mutations in GSD-Ib patients can be categorized into three distinct classes. First, substrate recognition is impaired by variants such as R28C/H, K29Q, K64Q, and K240Q, which compromise the positively charged binding cavity (Fig 2E), or by Y60C and F237S, which abolish essential hydrophobic interactions stabilizing the sugar moiety (Fig 2B and 2F).

This interpretation is supported by thermostability assays showing that mutations at these key coordinating residues (including R28, K29, Y60, K64, and K240) significantly reduce G6P-induced thermal shifts relative to the wild type (Fig 2G). Furthermore, these structural and thermostability defects directly translate to a severe loss of transport function, as demonstrated by the marked reduction in [^13^C_6_]G6P uptake for these mutants (Fig 2H). Second, the outward-open conformation is destabilized by mutations including D72N, T364M, R126C, T354K, T136M, and D285N, which break critical salt bridges and hydrogen bonds locking the NTD and CTD (Fig 2D). Third, the inward-facing state is compromised by variants such as D47N, K46E, K389E, A388P, T157I, and S263P, which prevent tight sealing of the ER luminal gate (Fig 3B and 3C). The remaining unmapped mutations likely impair overall structural integrity and folding and will require further investigation; together, these mechanistic classes provide a clear molecular rationale for the loss of function observed in patients.

During the preparation of this manuscript, independent studies describing SLC37A4 structures in various conformational states were published [21,22]. Our models exhibit remarkable convergence with these external datasets; notably, the overall structure of our SLC37A4-G6P complex is highly similar to those from the two previously published studies (S9A and S9B Fig), and the global architectures and the specific binding mode of CHA are virtually identical across all studies (S8 Fig).

In contrast to this conserved inhibitor binding, the precise coordination of G6P varies considerably among the models (S9C Fig). Interestingly, while the position of the H366 side chain remains largely unchanged across all three structures, its involvement in substrate binding differs dramatically. In our structure (SLC37A4-G6P), the phosphate group interacts with K64 and is close to Y60, but the glucose moiety points away, leaving H366 at a distance of 10.7 Å with no direct interaction (S9C Fig, left). Conversely, in PDB 9W1P, the substrate appears inverted, with the glucose ring forming close contacts (2.5–2.8 Å) with Y60, K64, and H366 (S9C Fig, middle). Meanwhile, in PDB 9KVV, the phosphate group remains near Y60 and K64 (similar to our model), but the glucose moiety is angled upwards to form a 3.3 Å interaction with H366 (S9C Fig, right). Because G6P in our structure lacks this additional coordination from H366, it exhibits a looser binding mode involving fewer overall protein contacts.

We interpret this loosely bound G6P in the outward-open conformation as representing a distinct intermediate state along the transport cycle, specifically capturing a transient moment of G6P release. Although the exact cause is unclear, subtle differences in sample preparation or lipid environments likely trapped these distinct conformational snapshots. Ultimately, despite these local variations in substrate coordination, the high consistency in overall architecture across independent investigations serves as a powerful cross-validation of our findings. This collective agreement establishes a definitive structural framework for SLC37A4, providing a highly reliable foundation for interpreting disease-associated mutations and guiding rational drug design for glycogen storage disease type Ib.

## Materials and methods

### Cloning, protein expression and purification

The cDNA encoding full length SLC37A4 was cloned into the pCAG vector with a C-terminal 3× Flag tag; all mutants were generated via standard PCR-based mutagenesis. For protein expression, HEK293F cells were maintained in suspension culture using SMM293-TII medium (Sino Biological) at 37°C under 8% CO_2_. When cell density reached 2 × 10^6^ cells/mL, the cultures were transfected with plasmid premixed with PEI at a 1:3 mass ratio. Cells were harvested by centrifugation at 2,000*g* 48 h post-transfection. The cell pellets were flash-frozen in liquid nitrogen and stored at −80 °C until further use.

All purification steps were performed at 4 ℃ or on ice. Cell pellets were thawed on ice-water bath and resuspended in Buffer A (25 mM HEPES pH 7.5, 150 mM NaCl, protease inhibitor cocktail containing 0.8 μM pepstatin A, 2 μM leupeptin, 2 μM aprotinin) and lysed by sonication on ice (10 s pulses, 20 s intervals, 30% amplitude). Membrane fractions were isolated by ultracentrifugation at 158,600*g* for 1 h. The membrane pellets were solubilized in Buffer B (25 mM HEPES pH 7.5, 150 mM NaCl, 1% (w/v) LMNG and 0.1% (w/v) cholesteryl hemisuccinate (CHS)). The insoluble material was removed by ultracentrifugation at 158,600*g* for 1 h. The clarified supernatant was incubated with anti-FLAG affinity resin (Smart-Lifesciences) for 1 hr. The resin was washed with 10 column volumes (CV) of Buffer C (25 mM HEPES pH 7.5, 150 mM NaCl, 0.005% [w/v] LMNG, 0.0005% [w/v] CHS). The proteins were eluted by Buffer C supplemented with 0.25 mg/mL 3× FLAG peptide (Sangon). The eluate was concentrated and further purified by gel filtration using Superdex 200 Increase 10/300 GL column (Cytiva) pre-equilibrated with Buffer D (25 mM HEPES pH 7.5, 150 mM NaCl, 0.005% LMNG, 0.0005% CHS). Peak fractions were collected and concentrated to 10–15 mg/mL for cryo-EM grid preparation.

For SLC37A4 nanodisc, MSP protein and lipid extract mixture were added to the FLAG resin and incubated overnight at 4 ℃. Detergent was removed by biobeads, protein was then eluted and further purified with gel filtration using Superdex 200 pre-equilibrated with Buffer E (25 mM HEPES pH 7.5, 150 mM NaCl). In parallel, an SLC37A4–GFP fusion construct was cloned and purified using the same expression and purification pipeline, and used for thermostability assays.

### Cryo-EM grid preparation

For cryo-EM sample preparation, freshly purified SLC37A4 at a concentration of 10–15 mg/mL was applied to glow-discharged UltrAufoil R1.2/1.3 Au 300-mesh holey carbon grids. For the ligand-bound samples, the protein was incubated with 100 mM G6P (for SLC37A4–G6P) or 10 mM CHA (for SLC37A4–CHA) for 1 hour on ice before grid preparation. Aliquots of 3 μl of sample were applied to the grids in a Vitrobot Mark IV (Thermo Fisher Scientific) operating at 4 °C and 100% relative humidity, blotted for 3.5 s with a blot force of 1, and rapidly plunge frozen into liquid ethane cooled by liquid nitrogen. Frozen grids were stored in liquid nitrogen until data collection.

### Cryo-EM data processing and structure refinement

For SLC37A4 apo data set, the Cryo-EM data were collected on Titan Krios microscope equipped with Falcon 4 camera. Motion correction was performed with patch motion correction in CryoSPARC to correct beam-induced motion [23]. Following motion correction, the contrast transfer function of micrographs was estimated. Micrographs with CTF fit resolution worse than 4 Å were discarded.

Initial particle picking was performed using blob pick with particle diameter of 80–120 Å. These particles were subjected to multiple rounds of 2D classification to generate high-quality 2D templates with visible secondary structure feature. These 2D class averages were used for template-based particle picking. To maximize the recovery of high-quality particles, the selected particles were used to train a convolutional neural network model using Topaz, which then picked additional particles missed by blob pick and template pick [24]. Particles from blob pick, template pick and Topaz were merged and duplicates were removed. The merged particles set underwent multiple rounds of 2D classification, only classes with clear secondary structure were kept for ab initio reconstruction and heterogeneous refinement. Given that SLC37A4 is a small membrane protein lacking a large soluble domain, a seeding strategy was employed in the heterogeneous refinement to enlarge particle pool [25]. Good particles were selected for NU-refinement, yielding a map with a resolution of 3.0 Å.

The particle coordinates were imported into Relion and the particles were re-extracted [26,27]. A final round of 3D auto-refinement was performed, followed by Bayesian polishing and CTF refinement, which improved the overall resolution to 2.8 Å. The same data processing strategy was applied to both the SLC37A-G6P and SLC37A-CHA datasets.

For model building, an initial structural model of SLC37A4 was obtained via AlphaFold prediction [28]. The predicted model was rigid-body fitted into the cryo-EM reconstruction map using UCSF chimera [29]. Regions of the predicted model that did not agree with the experimental density were manually rebuilt using COOT [30]. The model was finalized through iterative cycles of manual adjustment and automatic real-space refinement using Phenix (phenix.real_space_refine, applying secondary structure and geometry restraints) [31]. All representations of cryo-EM density and structural models were prepared with Chimera [29] and PyMOL [32]. A summary of data collection, processing, and model-refinement statistics for all three data sets is provided in Table 1.

### Thermal shift analysis

For thermal shift analysis of SLC37A4, a GFP-based assay was employed in which GFP was fused to the C-terminus of SLC37A4. The SLC37A4–GFP fusion proteins (wild-type and mutants) were purified using the same procedure described above. The GFP thermal shift assays were carried out according to previous studies [33,34]. Purified GFP-tagged SLC37A4 was diluted to 0.6 µM in assay buffer (20 mM HEPES pH 7.5, 150 mM NaCl, 1% (w/v) LMNG) in a final volume of 90 µl per well and incubated in the presence or absence of 10 mM G6P for 10 min. After incubation, 10 µL of 10% (w/v) β-n-octyl-β-d-glucopyranoside was added to each well. Samples were then heated to 10, 20, 30, 40, 50, 60, 70, or 80 ℃ for 10 min, centrifuged to remove aggregated protein, and the supernatant was transferred to a black 96-well plate. Soluble GFP fluorescence was measured on a plate reader (excitation 488 nm, emission 512 nm), and values were normalized to the signal at the lowest temperature condition. To generate thermal transition curves, the fluorescence data were normalized by setting the highest and lowest data points to 100% and 0%, respectively. The normalized fluorescence values were plotted against temperature, and apparent melting temperatures (Tm) for each condition were determined by fitting the data to a sigmoidal four-parameter logistic function using GraphPad Prism. To evaluate whether the mutated residues affected G6P binding, the shift in melting temperature (ΔTm) was calculated for each protein by subtracting the Tm of the apo state from the Tm of the G6P-incubated state.

### [¹³C]G6P uptake assay in G6PT-expressing cells

For the cellular G6P uptake assay, the assay was preformed based on previously described method [22] with minor modification. HEK293F cell were transfected with SLC37A4 plasmids (WT or mutants) premixed with PEI at 1:3 mass ratio as described in the protein purification section. Forty-eight hours post-transfection, cells were pelleted by centrifugation at 1,000*g* for 5 min, washed once with Wash Buffer A1 (1× Hanks’ Balanced Salt Solution [HBSS, Gibco] supplemented with 2% FBS), and permeabilized with Permeabilization Buffer A2 (1× HBSS supplemented with 10 µM digitonin) at 37 °C for 10 min. This low concentration of digitonin was utilized to selectively permeabilize the cholesterol-rich plasma membrane while maintaining the integrity of the ER membrane. Following permeabilization, cells were washed with 1× HBSS twice to remove residual buffer. Uptake was initiated by adding a 1:1 molar mixture of [¹³C_6_]G6P and unlabeled G6P (MedChemExpress) to a final concentration of 1 mM. After a 10-min incubation at 37 °C, the reaction was quenched by washing the cells twice with 500 µL of ice-cold 1× HBSS. Intracellular [¹³C_6_]G6P was extracted using 200 µL of pre-chilled methanol: acetonitrile (2:1, v/v), followed by ultrasonication at 4 °C for 10 min. The extract was centrifuged at 12,000 rpm for 20 min at 4 °C, and 150 µL of the resulting supernatant was transferred to a fresh tube and lyophilized for subsequent quantitative LC–MS analysis. The remaining cell pellet was lysed in 400 µL of 0.1 M KOH, and the protein concentration was determined using a BCA protein assay kit (Yeasen). [¹³C_6_]G6P uptake levels were normalized to the corresponding total protein content. All experiments were performed at least in triplicate, and data were analyzed using nonlinear regression in Prism 8.0.1 (GraphPad Software).

### Quantification of [¹³C_6_]G6P by LC–MS

Lyophilized [¹³C_6_]G6P samples were quantified using liquid chromatography–mass spectrometry (LC–MS). Samples were resolved on an Agilent 1290 Infinity II-6495B UPLC–MS system equipped with an ACQUITY UPLC BEH Amide column (2.1 mm × 100 mm, 1.7 μm). The mobile phase consisted of acetonitrile:water (9:1, v/v) supplemented with 15 mM ammonium acetate and 0.3% ammonium hydroxide. Detection was performed in negative electrospray ionization mode (ESI−), and [¹³C_6_]G6P was monitored via multiple reaction monitoring (MRM) at *m*/*z* 264.9. Data acquisition and analysis were performed using MassHunter Workstation Data Acquisition (version 10.0 SR1) and Quantitative Analysis (version 10.0) software (Agilent Technologies), respectively. Compound identity was confirmed by matching retention times and MRM transitions to a reference standard. Quantification was achieved using an external calibration curve, with final concentrations calculated based on sample volume.

### Western blot

HEK293F cells (1 × 10^6^ cells/mL) expressing empty vector, wild-type G6PT, or mutants were harvested, washed with PBS, and lysed in 400 μL buffer (20 mM HEPES pH 7.5, 150 mM NaCl, 1% [w/v] DDM, 0.1% [w/v] CHS, 2 µM pepstatin A, 4.2 µM leupeptin, and 0.8 µM aprotinin). After 1.5 h at 4 °C, lysates were cleared by ultracentrifugation. Solubilized proteins were resolved by SDS-PAGE and transferred to PVDF membranes. Membranes were blocked for 1 h at room temperature in TBST containing 5% nonfat milk and then incubated overnight at 4 °C with rabbit monoclonal anti-Flag antibody (1:3,000; Cell Signaling Technology, Cat#14793) and anti-calnexin antibody (1:1,000; Cell Signaling Technology, Cat#2679S), which served as an internal loading control. After washing, membranes were incubated with HRP-conjugated secondary antibodies for 45 min at room temperature. Protein bands were visualized by chemiluminescence using an iBright FL1500 imaging system.

## Supporting information

S1 Fig Purification and reconstitution of human SLC37A4. **(A)** Size-exclusion chromatography (SEC) profile and SDS-PAGE of SLC37A4 purified in LMNG detergent. **(B)** SEC profile and SDS-PAGE of SLC37A4 reconstituted into MSP1E1D1 nanodiscs.
(TIF)

S2 Fig Cryo-EM data processing and validation of human SLC37A4-apo. **(A)** Workflow of cryo-EM data processing, including representative micrographs, 2D class averages showing dimers and monomers, 3D classification, and refinement steps leading to the final reconstruction. **(B)** Angular distribution of particles used in the final reconstruction. **(C)** Fourier shell correlation (FSC) curves indicating the overall resolution at 0.143 cutoff (blue) and map-to-model correlation (red). **(D)** Local resolution map showing resolution distribution across the density map. **(E)** Representative cryo-EM densities for transmembrane helices (TM1–TM12) with the atomic model fitted.
(TIF)

S3 Fig Cryo-EM data processing and validation of human SLC37A4-G6P. **(A)** Workflow of cryo-EM data processing, including representative micrographs, 2D class averages showing dimers and monomers, 3D classification, and refinement steps leading to the final reconstruction. **(B)** Angular distribution of particles used in the final reconstruction. **(C)** Fourier shell correlation (FSC) curves indicating the overall resolution at 0.143 cutoff (blue) and map-to-model correlation (red). **(D)** Local resolution map showing resolution distribution across the density map. **(E)** Representative cryo-EM densities for transmembrane helices (TM1–TM12) with the atomic model fitted.
(TIF)

S4 Fig Cryo-EM density around the G6P pocket. Overlay of the SLC37A4–G6P (blue) and apo (orange) maps, both contoured at 3σ. The comparison reveals additional density for the bound G6P molecule near residues Y60, K64, and F237.
(TIF)

S5 Fig Thermal transition curves of wild-type and mutant SLC37A4. **(A–H)** GFP-based thermal shift assays for wild-type (WT) SLC37A4 (A) and mutants R28A (B), R28C (C), R28H (D), K29A (E), Y60A (F), K64A (G), and K240A (H). The normalized fluorescence intensity (FI) signal is plotted as a function of temperature. Assays were performed in the absence (gray circles) or presence (red triangles) of 10 mM glucose-6-phosphate (G6P). Data were from at least triplicates. The calculated apparent melting temperatures (Tm) for each individual replicate are indicated in each panel. The underlying data for this figure can be found in S1 Data.
(TIF)

S6 Fig Protein expression levels of SLC37A4 wild-type and mutants. Western blot analysis evaluating the expression of Flag-tagged wild-type (WT) and mutant SLC37A4 constructs (R28A, R28C, R28H, K29A, Y60A, K64A, and K240A). Calnexin was probed as a loading control. The results demonstrate that all generated mutants express at levels comparable to the WT protein, confirming that the observed reductions in transport activity are due to functional impairments rather than decreased protein stability or expression. The control lane represents the untransfected/empty vector negative control.
(TIF)

S7 Fig Cryo-EM data processing and validation of human SLC37A4-CHA. **(A)** Workflow of cryo-EM data processing, including representative micrographs, 2D class averages showing dimers and monomers, 3D classification, and refinement steps leading to the final reconstruction. **(B)** Angular distribution of particles used in the final reconstruction. **(C)** Fourier shell correlation (FSC) curves indicating the overall resolution at 0.143 cutoff (blue) and map-to-model correlation (red). **(D)** Local resolution map showing resolution distribution across the density map. **(E)** Representative cryo-EM densities for transmembrane helices (TM1–TM12) with the atomic model fitted.
(TIF)

S8 Fig Structural comparison of the inward-facing SLC37A4–CHA complex. Alignment of our SLC37A4–CHA structure (cyan) with a previously reported CHA-bound structure (PDB 9KV0, magenta). Orthogonal views highlight the conserved inward-facing architecture and overlapping CHA-binding sites.
(TIF)

S9 Fig Structural comparison of G6P-bound SLC37A4. **(A, B)** Orthogonal views aligning our SLC37A4–G6P structure (cyan) with previously reported G6P-bound models (PDB 9W1P, magenta; PDB 9KVV, green). **(C)** Detailed comparison of the G6P binding pockets across the three structures. Key interacting residues (Y60, K64, and H366) and the G6P ligand are shown as sticks, with measurement distances indicated in angstroms (Å).
(TIF)

S1 Raw Images Original uncropped and minimally adjusted gel and blot images corresponding to. S1A, S1B, and S6 Figs. The upper-left and upper-right panels correspond to S1A and S1B Fig, respectively; the lower panels correspond to S6 Fig. Dashed boxes indicate the regions cropped and displayed in the corresponding Supporting information panels.
(DOCX)

S1 Data Underlying numerical data for Figs 2G, 2H, and S5. This file contains individual replicate values, calculated means, standard deviations, and replicate numbers used to generate the plotted data. Replicate values are arranged vertically, and summary tables indicate how the plotted mean ± s.d. values were derived.
(XLSX)

## Abbreviations

CHA: chlorogenic acid
CHS: cholesteryl hemisuccinate
cryo-EM: cryo-electron microscopy
CTD: C-terminal domain
CV: column volumes
ER: endoplasmic reticulum
ESI: electrospray ionization mode
FSC: Fourier shell correlation
G6P: glucose-6-phosphate
G6PT: glucose-6-phosphate transporter
GSD Ib: glycogen storage disease type Ib
HBSS: Hanks’ Balanced Salt Solution
LC–MS: liquid chromatography–mass spectrometry
LMNG: lauryl maltose neopentyl glycol
MFS: Major Facilitator Superfamily
MRM: multiple reaction monitoring
NTD: N-terminal domain; Pi, inorganic phosphate
RMSD: root-mean-square deviation
TMs: transmembrane helices
