# Supplementary figures and images for: Structural basis for substrate recognition and inhibition of human glucose-6-phosphate transporter SLC37A4
Source: PLoS Biol. 2026 Jul 28;24(7):e3003833. doi: 10.1371/journal.pbio.3003833 (PMC13411879; doi:10.1371/journal.pbio.3003833)

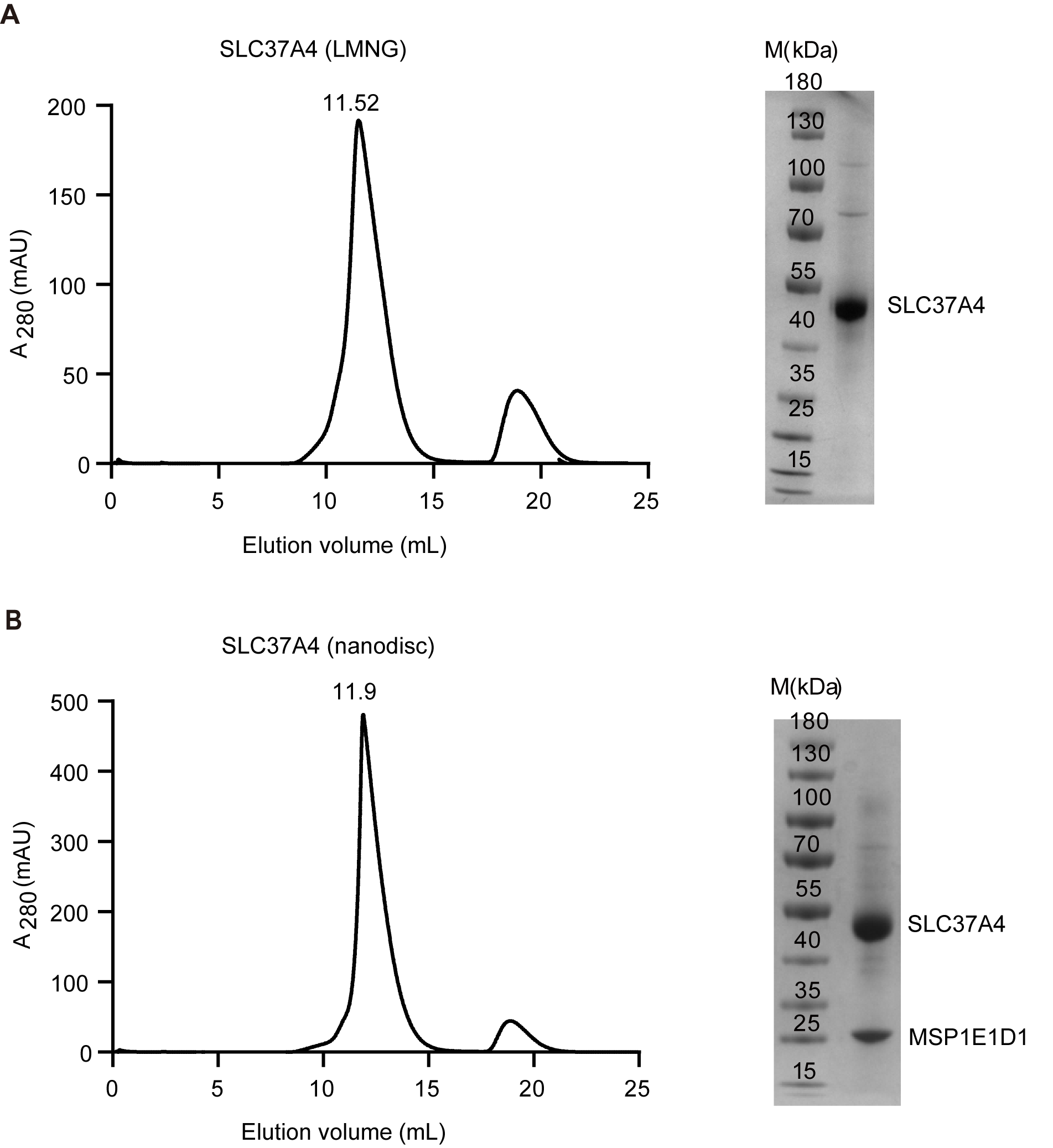

Supplement: S1 Fig — (A) Size-exclusion chromatography (SEC) profile and SDS-PAGE of SLC37A4 purified in LMNG detergent. (B) SEC profile and SDS-PAGE of SLC37A4 reconstituted into MSP1E1D1 nanodiscs. (TIF) [file pbio.3003833.s001.tif]

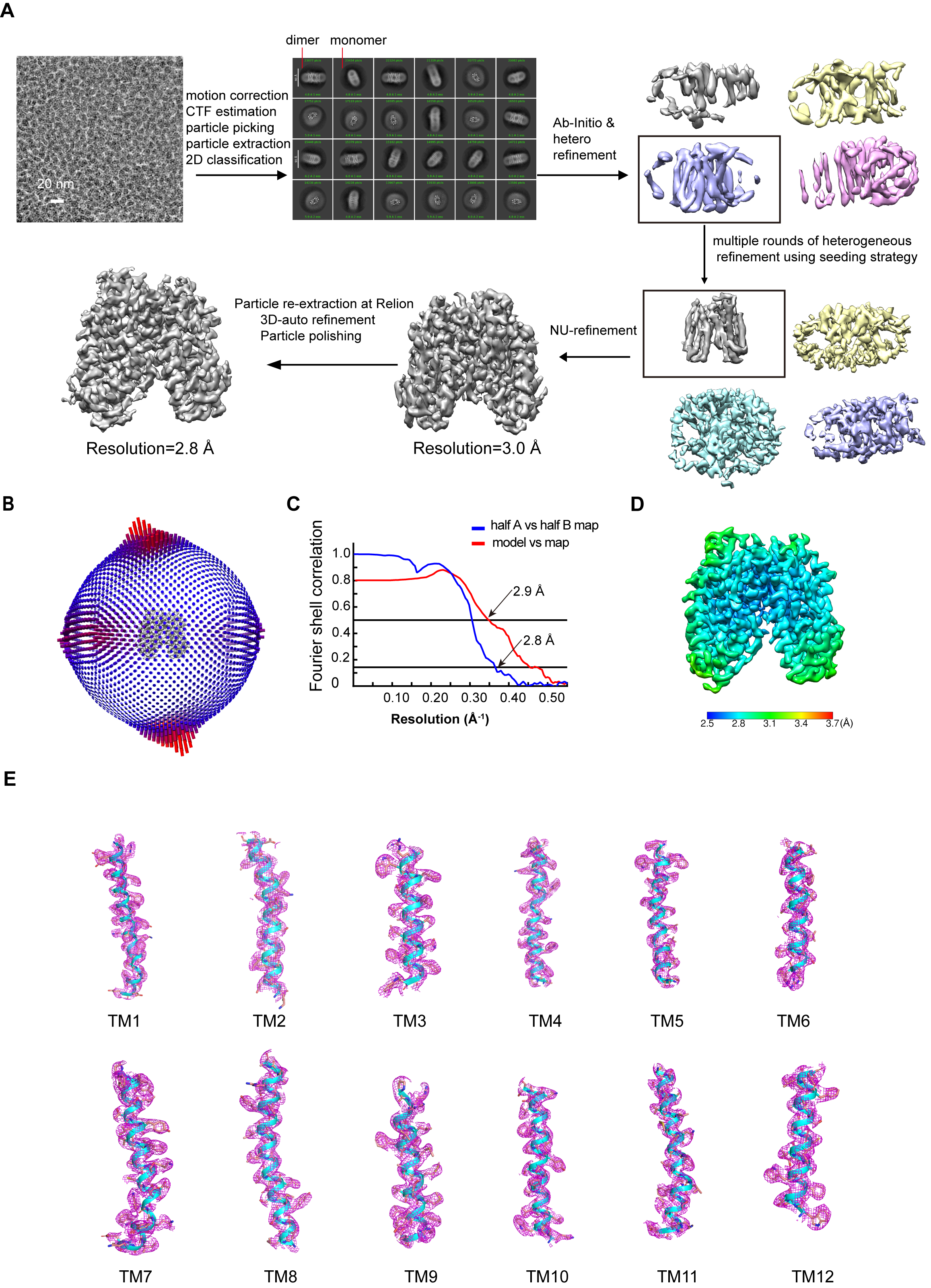

Supplement: S2 Fig — (A) Workflow of cryo-EM data processing, including representative micrographs, 2D class averages showing dimers and monomers, 3D classification, and refinement steps leading to the final reconstruction. (B) Angular distribution of particles used in the final reconstruction. (C) Fourier shell correlation (FSC) curves indicating the overall resolution at 0.143 cutoff (blue) and map-to-model correlation (red). (D) Local resolution map showing resolution distribution across the density map. (E) Representative cryo-EM densities for transmembrane helices (TM1–TM12) with the atomic model fitted. (TIF) [file pbio.3003833.s002.tif]

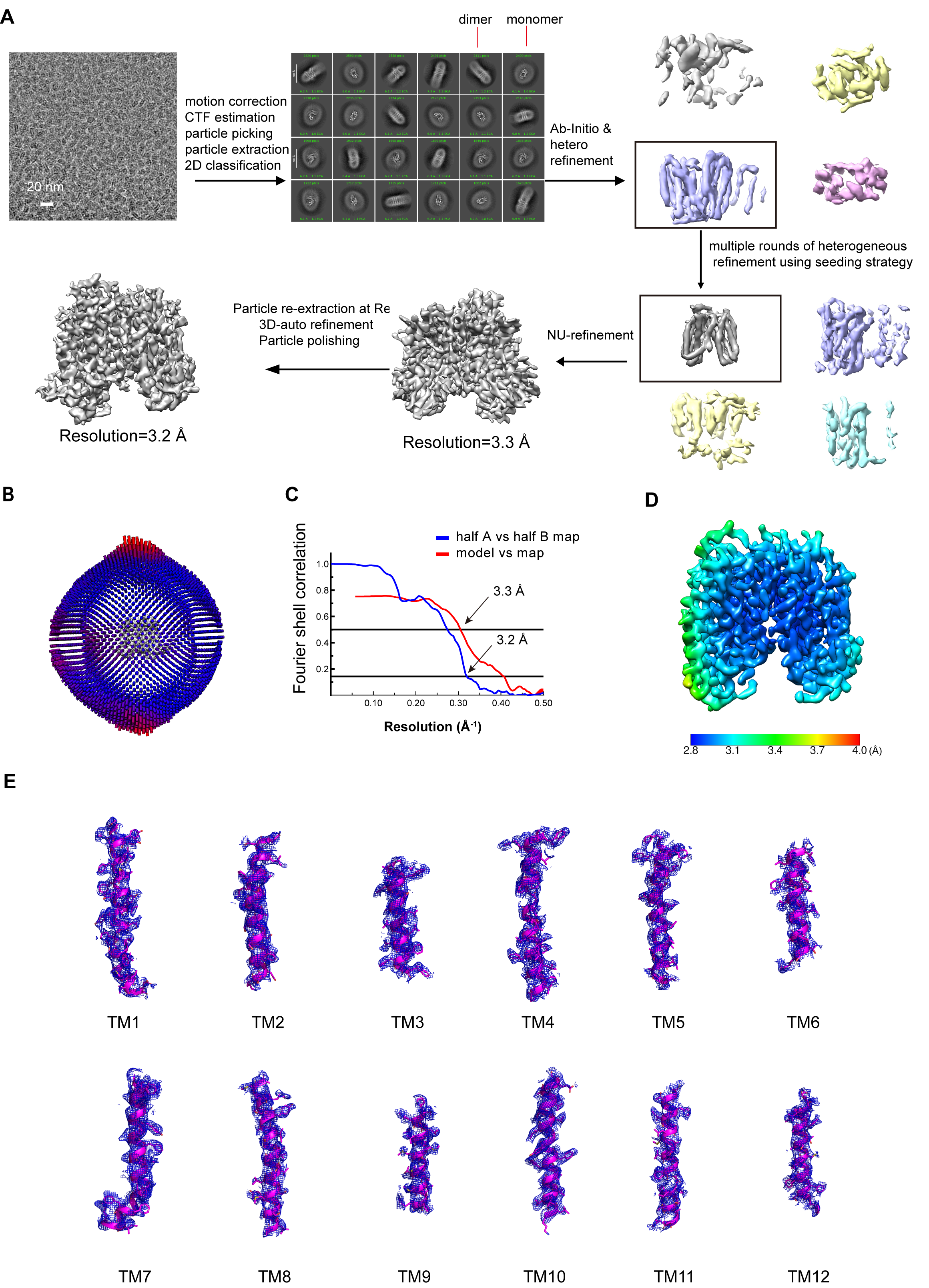

Supplement: S3 Fig — (A) Workflow of cryo-EM data processing, including representative micrographs, 2D class averages showing dimers and monomers, 3D classification, and refinement steps leading to the final reconstruction. (B) Angular distribution of particles used in the final reconstruction. (C) Fourier shell correlation (FSC) curves indicating the overall resolution at 0.143 cutoff (blue) and map-to-model correlation (red). (D) Local resolution map showing resolution distribution across the density map. (E) Representative cryo-EM densities for transmembrane helices (TM1–TM12) with the atomic model fitted. (TIF) [file pbio.3003833.s003.tif]

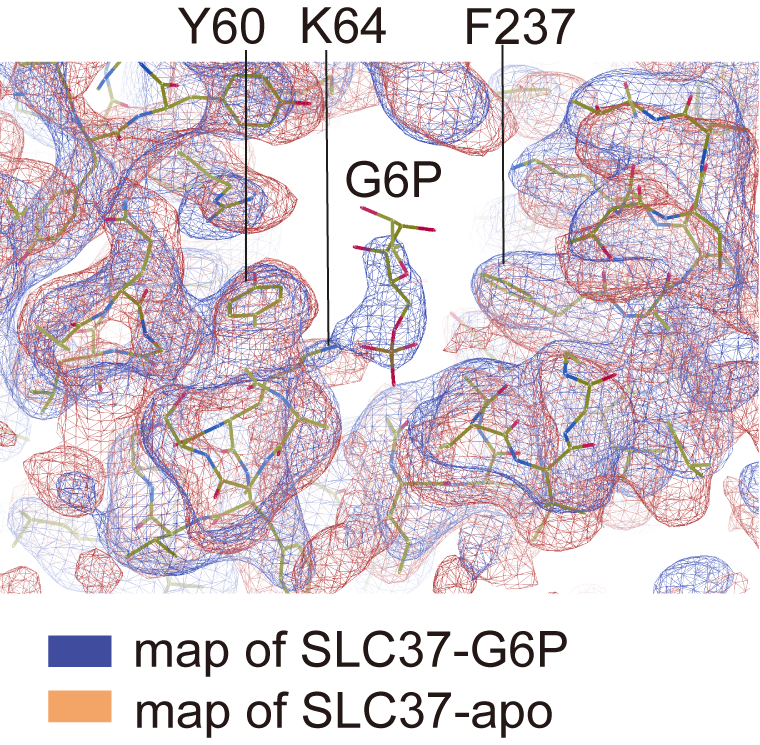

Supplement: S4 Fig — Overlay of the SLC37A4–G6P (blue) and apo (orange) maps, both contoured at 3σ. The comparison reveals additional density for the bound G6P molecule near residues Y60, K64, and F237. (TIF) [file pbio.3003833.s004.tif]

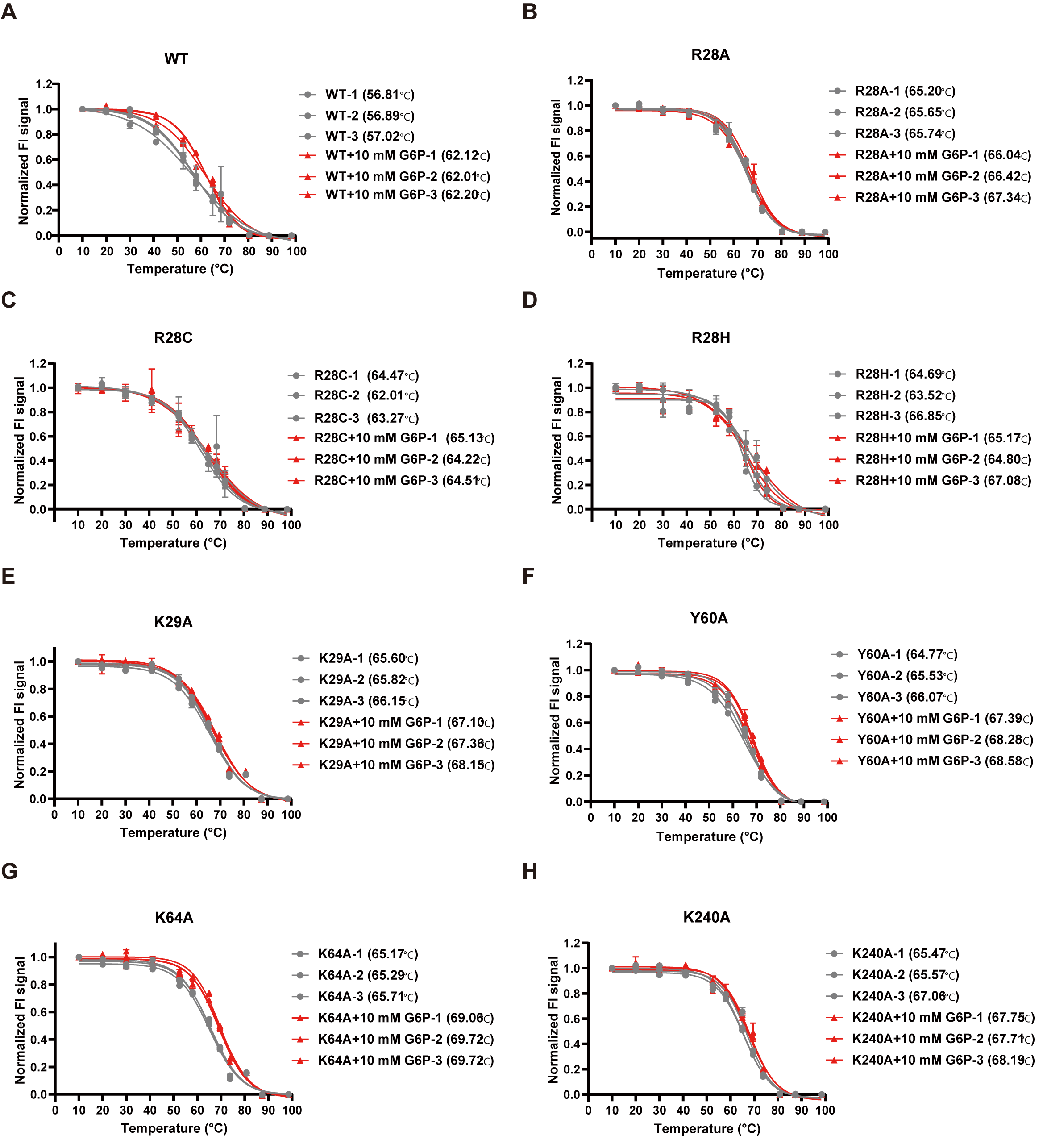

Supplement: S5 Fig — (A–H) GFP-based thermal shift assays for wild-type (WT) SLC37A4 (A) and mutants R28A (B), R28C (C), R28H (D), K29A (E), Y60A (F), K64A (G), and K240A (H). The normalized fluorescence intensity (FI) signal is plotted as a function of temperature. Assays were performed in the absence (gray circles) or presence (red triangles) of 10 mM glucose-6-phosphate (G6P). Data were from at least triplicates. The calculated apparent melting temperatures (Tm) for each individual replicate are indicated in each panel. The underlying data for this figure can be found in S1 Data. (TIF) [file pbio.3003833.s005.tif]

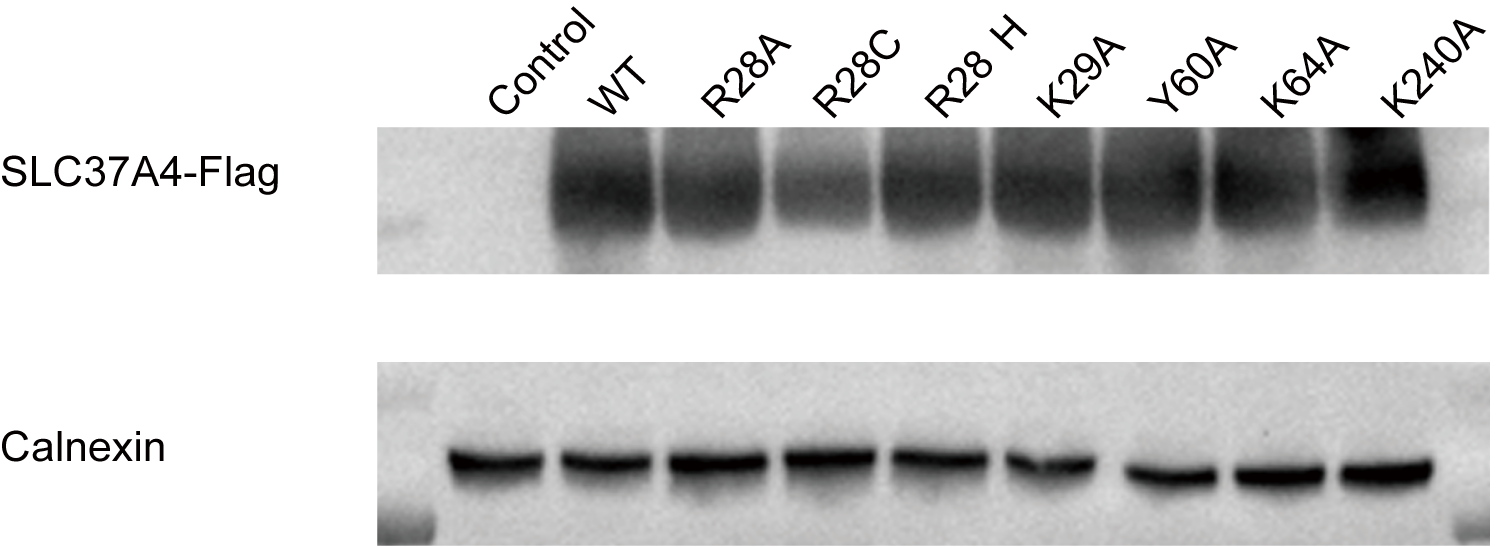

Supplement: S6 Fig — Western blot analysis evaluating the expression of Flag-tagged wild-type (WT) and mutant SLC37A4 constructs (R28A, R28C, R28H, K29A, Y60A, K64A, and K240A). Calnexin was probed as a loading control. The results demonstrate that all generated mutants express at levels comparable to the WT protein, confirming that the observed reductions in transport activity are due to functional impairments rather than decreased protein stability or expression. The control lane represents the untransfected/empty vector negative control. (TIF) [file pbio.3003833.s006.tif]

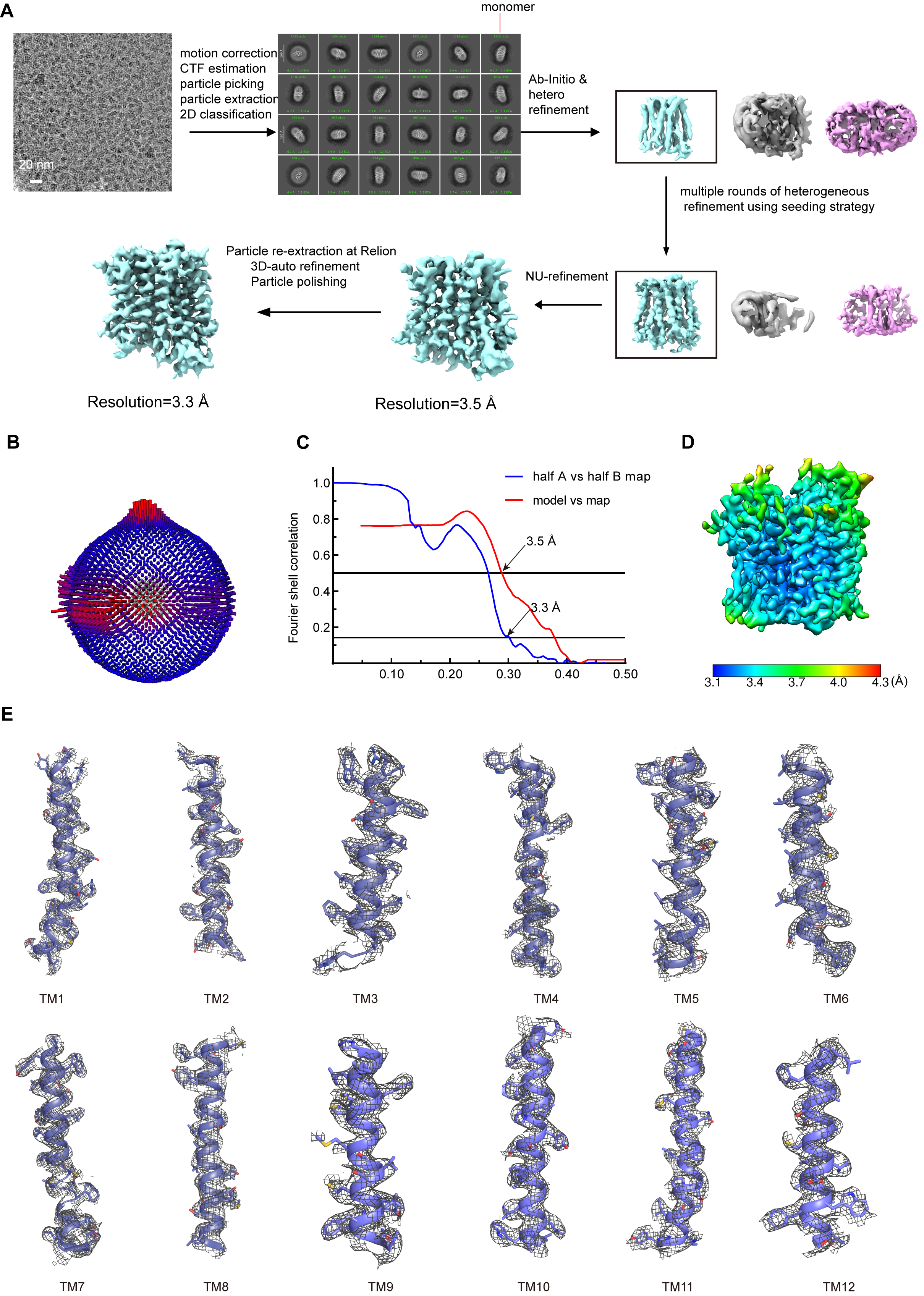

Supplement: S7 Fig — (A) Workflow of cryo-EM data processing, including representative micrographs, 2D class averages showing dimers and monomers, 3D classification, and refinement steps leading to the final reconstruction. (B) Angular distribution of particles used in the final reconstruction. (C) Fourier shell correlation (FSC) curves indicating the overall resolution at 0.143 cutoff (blue) and map-to-model correlation (red). (D) Local resolution map showing resolution distribution across the density map. (E) Representative cryo-EM densities for transmembrane helices (TM1–TM12) with the atomic model fitted. (TIF) [file pbio.3003833.s007.tif]

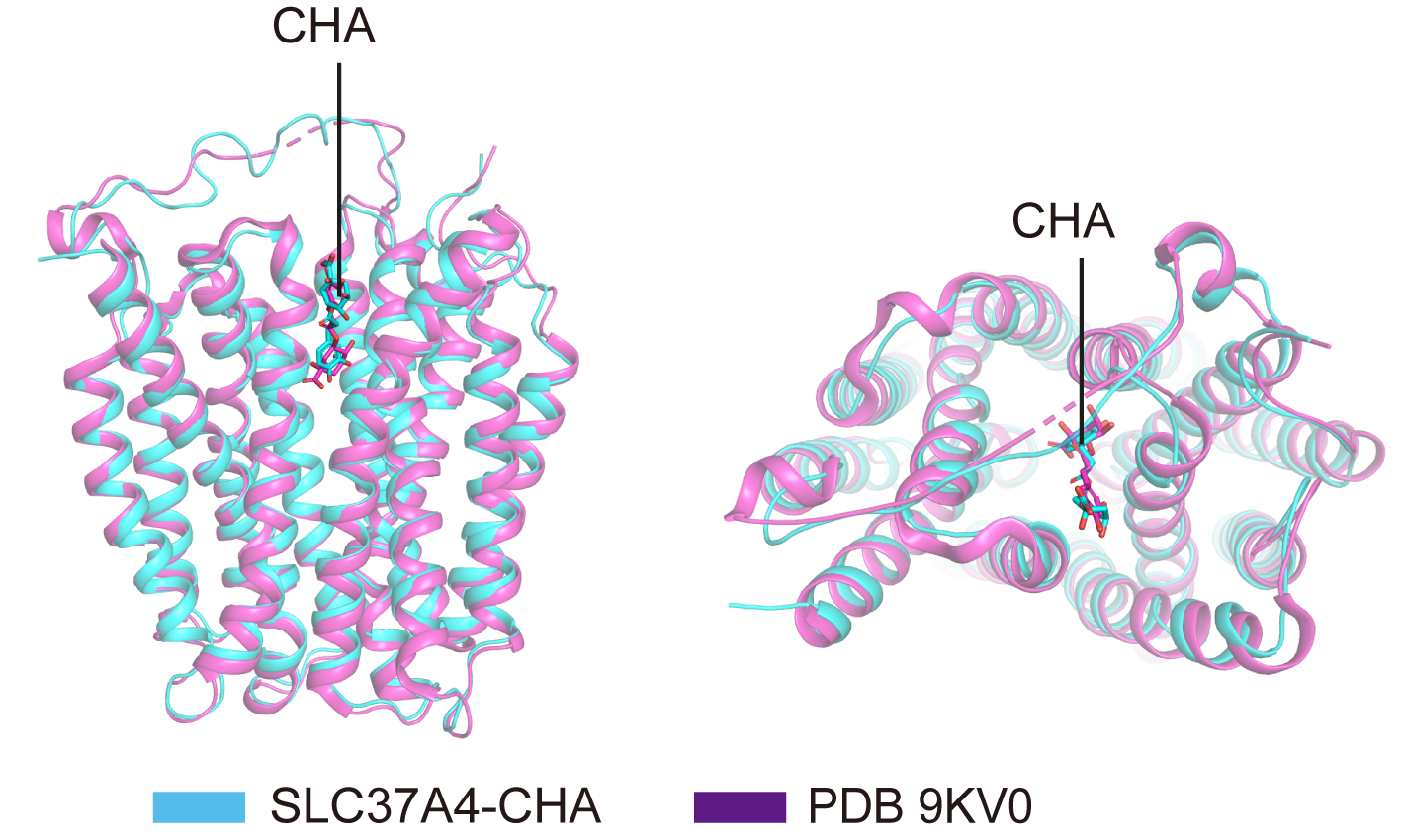

Supplement: S8 Fig — Alignment of our SLC37A4–CHA structure (cyan) with a previously reported CHA-bound structure (PDB 9KV0, magenta). Orthogonal views highlight the conserved inward-facing architecture and overlapping CHA-binding sites. (TIF) [file pbio.3003833.s008.tif]

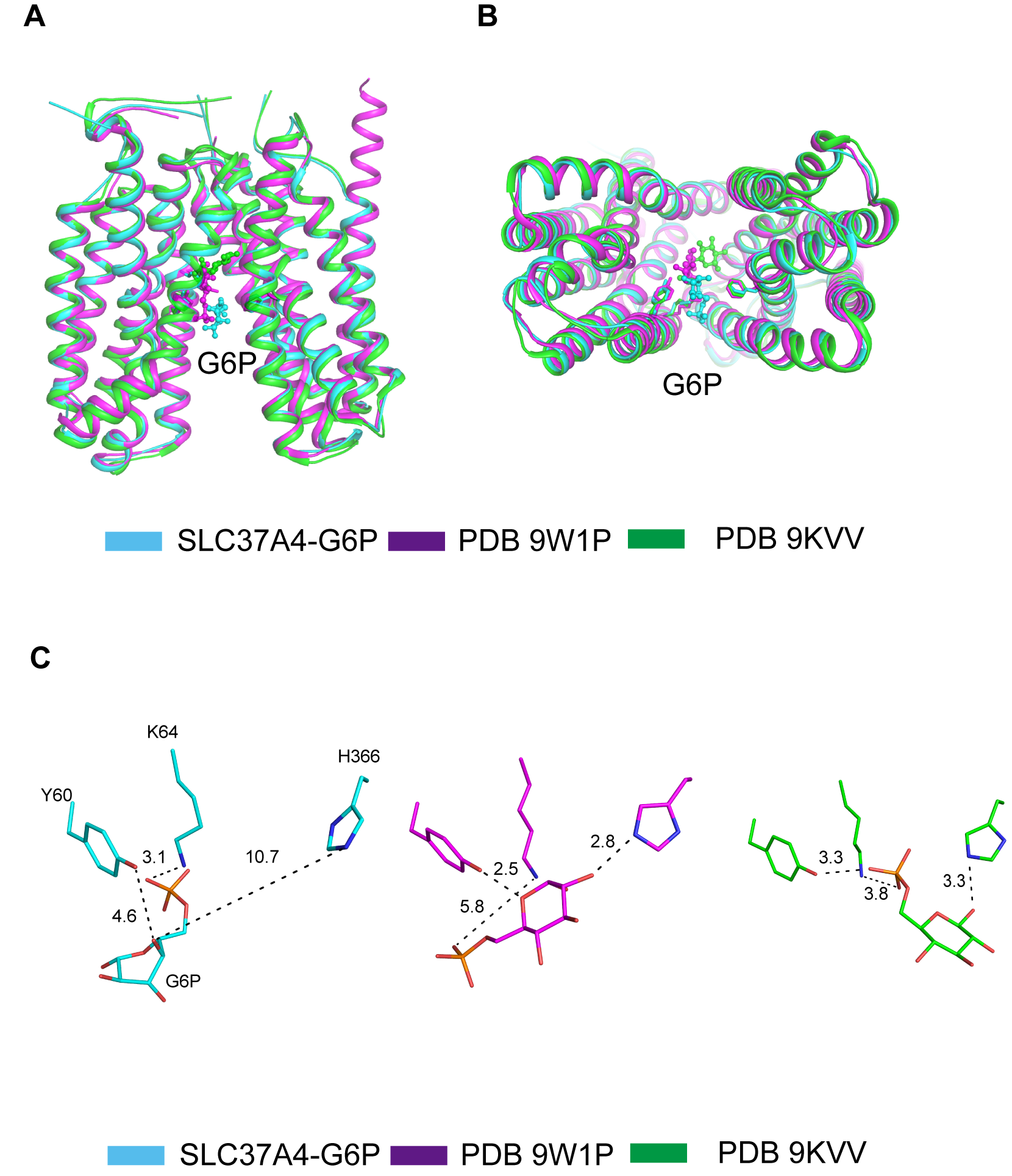

Supplement: S9 Fig — (A, B) Orthogonal views aligning our SLC37A4–G6P structure (cyan) with previously reported G6P-bound models (PDB 9W1P, magenta; PDB 9KVV, green). (C) Detailed comparison of the G6P binding pockets across the three structures. Key interacting residues (Y60, K64, and H366) and the G6P ligand are shown as sticks, with measurement distances indicated in angstroms (Å). (TIF) [file pbio.3003833.s009.tif]
