# Supplementary material for: Structural basis for substrate recognition and inhibition of human glucose-6-phosphate transporter SLC37A4
Source: PLoS Biol. 2026 Jul 28;24(7):e3003833. doi: 10.1371/journal.pbio.3003833 (PMC13411879; doi:10.1371/journal.pbio.3003833)
Supplement: S1 Raw Images — S1A, S1B, and S6 Figs. The upper-left and upper-right panels correspond to S1A and S1B Fig, respectively; the lower panels correspond to S6 Fig. Dashed boxes indicate the regions cropped and displayed in the corresponding Supporting information panels. (DOCX) [file pbio.3003833.s010.docx]

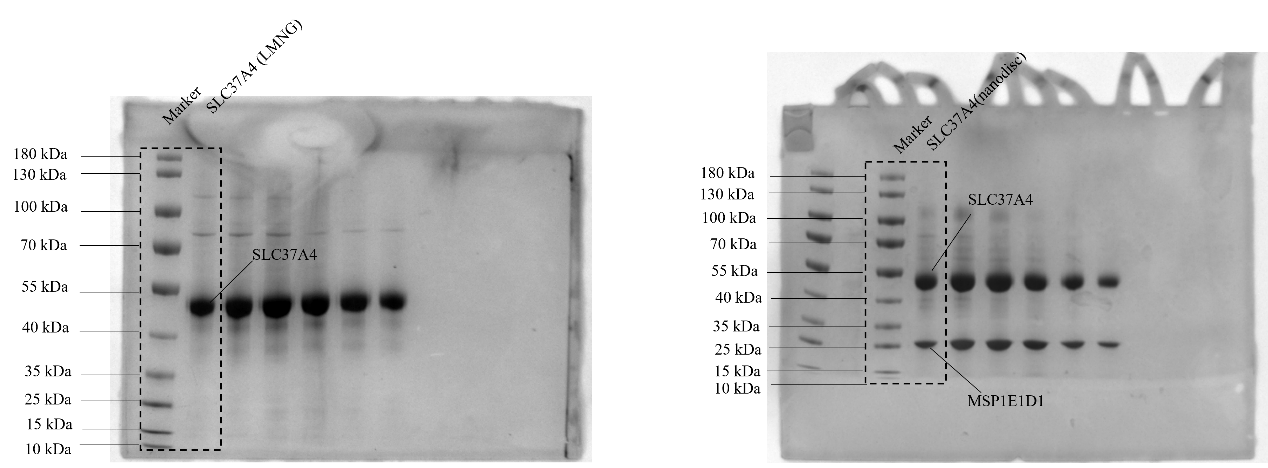


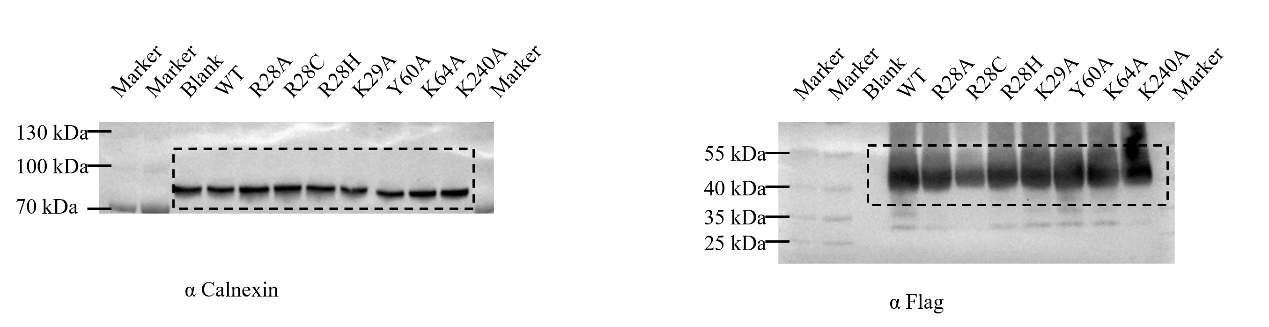


**S1 Raw Images.** Original uncropped and minimally adjusted gel and blot images corresponding to S1A, S1B, and S6 Figs. The upper-left and upper-right panels correspond to S1A and S1B Fig, respectively; the lower panels correspond to S6 Fig. Dashed boxes indicate the regions cropped and displayed in the corresponding Supporting Information panels.
