# Supplementary material for: Structural basis for substrate recognition and inhibition of human glucose-6-phosphate transporter SLC37A4
Source: PLoS Biol. 2026 Jul 28;24(7):e3003833. doi: 10.1371/journal.pbio.3003833 (PMC13411879; doi:10.1371/journal.pbio.3003833)
Supplement: Attachment — Submitted filename: response-041026.docx [file pbio.3003833.s014.docx]

**Reviewer #1**: In this work, the authors report the cryo-EM structures of human SLC37A4 in apo, G6P-bound, and CHA-bound states, providing an important structural framework for substrate recognition and inhibition. The manuscript is well written. Although a few highly related studies have been published recently, considering the scope of PLOS Biology, this work remains valuable and should be suitable for publication if the following concerns are properly addressed.

1. Transport assays SLC37A4 is a G6P/Pi exchanger, the mechanistic conclusions would be substantially strengthened by adding a transport assay (e.g., using reconstituted proteoliposomes or a cellular transport assay). Some mutational analysis using a transport assay should be performed in the revision.

**Response:** We sincerely thank the reviewer for this excellent suggestion, which has substantially strengthened our mechanistic conclusions.

To functionally validate our structural findings, we performed transport assays using digitonin-permeabilized cells overexpressing wild-type (WT) or structure-guided mutants of SLC37A4. We have revised Fig. 2 (shown below) to include these new transport activity data. As shown in the newly added Figure 2h, WT SLC37A4 exhibits robust [^13^C]G6P uptake, whereas mutations at key substrate-binding residues (e.g., R28A, K29A, and Y60A) significantly impair transport activity.

These functional data directly validate our structural model for substrate recognition. We have updated the Results and Methods sections in the revised manuscript to detail these experiments and thank the reviewer again for prompting this valuable addition.

**Fig 2. Structural basis of G6P recognition by SLC37A4.**

(a) Superposition of the SLC37A4 apo state (grey) and the G6P-bound state (NTD in yellow, CTD in cyan). The structures are shown as cylindrical cartoons viewed along the membrane plane. The bound G6P molecule is shown as red sticks in the central cavity.

(b) View of the superposed apo and G6P‑bound structures from the ER‑lumen side along the membrane normal, highlighting G6P and nearby residues R28, K29, Y60, K64, F237 and K240.

(c) Cryo-EM density of the bound G6P molecule, with a contour level of 3 σ.

(d) View of the interface between the N‑terminal and C‑terminal domains from the cytosolic side along the membrane normal, showing gating residues at the domain interface, including D72, R126, T136, D285, T354 and T364.

(e) Electrostatic surface representation of SLC37A4 viewed from the same orientation in b.

(f) Two‑dimensional schematic of the G6P‑binding sites, depicting hydrogen‑bond interactions between G6P and K64, as well as hydrophobic contacts with F237 and Y60.

(g) GFP-based thermostability assay showing G6P induced changes in melting temperature (ΔTm) for wild type (WT) SLC37A4 and G6P binding–site mutants (R28A, R28C, R28H, K29A, Y60A, K64A, K240A). Bars represent mean ± s.d. from at least three technical replicates.
(h) Relative [^13^C]G6P uptake for WT and mutant SLC37A4. Bars represent mean ± s.d. from at least three technical replicates.

2. Terminology for CHA as "allosteric"

The authors describe CHA as binding to an "allosteric" site. However, the structures show that the CHA-interacting region partially overlaps with the G6P-binding region. Therefore, it is unclear whether it is appropriate to refer to this site as allosteric. Thus, if the authors continue to use the term "allosteric," please provide a more detailed explanation and justification. Otherwise, please refrain from using "allosteric".

**Response:** We thank the reviewer for this careful observation. We originally used the term 'allosteric' because, in our specific G6P-bound structure, residue H366 does not directly contact the substrate, making the CHA and G6P binding sites appear spatially distinct. However, in our CHA-bound structure, H366 directly interacts with the inhibitor. Furthermore, as the reviewer rightly points out and recently published structures confirm (PMIDs: 41136424 and 41225049), H366 acts as a shared interaction node that coordinates both G6P and CHA.

Because these binding pockets physically overlap at this key residue, we completely agree that designating the CHA site as 'allosteric' is misleading. Accordingly, we have removed the term 'allosteric' throughout the revised manuscript. To explicitly illustrate this overlap, we have also included a structural comparison of our models with the recently published structures in the revised manuscript (Supplementary Fig 9c).

**Supplementary Figure 9. Structural comparison of G6P-bound SLC37A4.**
**(a, b)** Orthogonal views aligning our SLC37A4–G6P structure (cyan) with previously reported G6P-bound models (PDB 9W1P, magenta; PDB 9KVV, green).
**(c)** Detailed comparison of the G6P binding pockets across the three structures. Key interacting residues (Y60, K64, and H366) and the G6P ligand are shown as sticks, with measurement distances indicated in angstroms (Å).

3. TyposPage 17, Line 16 (Data Availability)"SCL37A4" should be corrected as "SLC37A4". "EMD-64869" should be corrected as "EMD-67869". Supplementary Figure 5. In panel d, "Y64" should be corrected as "Y60". In panel e, "PDB 9KVO" should be corrected as "PDB 9KV0".

**Response:** We sincerely thank the reviewer for their careful reading of our manuscript. We have corrected all the specified typos in the text (Page 17) and in Supplementary Figure 7 (formerly Supplementary Figure 5e-f).

**Reviewer #2**: In this manuscript, Li et al. report cryo-EM structures of the human glucose-6-phosphate transporter (G6PT/SLC37A4) in the apo and G6P-bound states, as well as in complex with the allosteric inhibitor chlorogenic acid (CHA). These structures capture the transporter in both inward- and outward-facing conformations characteristic of Major Facilitator Superfamily transporters, allowing them to draw conclusions about the conformational changes occurring during the transport cycle. Overall, the study provides new insights into the structure, function, and conformational dynamics of the SLC37 family. The structural work is of high quality, but I have several comments that may help to further strengthen the manuscript.

1.) Modeling of the cytoplasmic loop connecting the N- and C-bundle. The authors did not specify which portion of the cytoplasmic loop connecting TM6 and TM7 was modeled. A clear description and labeling in the figures would be helpful. Are there any disease related mutations located?

**Response**: We thank the reviewer for highlighting this point. We have revised the manuscript to explicitly state that the cytosolic loop connecting the N- and C-bundles (between TM6 and TM7) is highly flexible. As a result, the central portion of this loop (residues 202–211) lacked sufficient electron density and was omitted from the final model. To better illustrate this, we have added explicit labels and a dashed line representing the unmodeled region in revised Fig. 1b (shown below). Finally, a comprehensive literature search confirmed the absence of any known disease-related mutations within this specific region.

**Fig 1. Overall structure of human SLC37A4 in the apo state**

(a) Cryo‑EM density map of SLC37A4‑apo viewed along the membrane plane. The protein density is shown in green and detergent micelle shown in transparent grey.

(b) Cartoon representation of SLC37A4 structure in the same membrane plane orientation as in a. N-terminal domain NTD is shown in yellow and C-terminal domain CTD is shown in cyan, individual transmembrane helices are numbered. The dashed line indicates an unresolved cytosolic loop between residues 201 and 212 that connects TM6 and TM7, linking the NTD and CTD.

(c) View of SLC37A4 from the ER‑lumen side along membrane normal, obtained by a 90° rotation of the model in b, showing the arrangement of the NTD and CTD.

(d) Overall structure of SLC37A4 in the orientation in a, highlighting 4 positively charged residues in the putative substrate‑binding pocket.

(e) Cutaway view in the orientation of a, showing a slice through the putative substrate binding pocket.

(f) Electrostatic surface representation of SLC37A4 viewed from the same orientation in c, ER-lumen side along the membrane normal, showing the putative substrate‑binding pocket and the location of R28, K29, K64 and K240.

2.) Comments to figures

- all figures (main plus supplementary) illustrating EM densities require a statement on the shown contour level- label termini (at least in the first figure); loops or parts not modelled should be shown in dashed lines - enlarge the density for G6P in figure 2 and show from at least two different orientations- highlight EM density for CHA in figure 3 (not only in supplementary figure)

**Response:** We thank the reviewer for these constructive suggestions, which have significantly improved the clarity of our structural data presentation. We have updated all relevant figures and their corresponding legends accordingly. Specifically, we have added the contour levels for the displayed cryo-EM densities to the figure legends, noting that the G6P density is contoured at 3σ and the CHA density at 8σ. In the structural figures, we have now labeled the termini and indicated unmodeled loops with dashed lines. Furthermore, we have enlarged the G6P density in Figure 2c to display it from two different orientations. We have also incorporated the EM density for CHA directly into the main text in Figure 3f. Consequently, to avoid redundancy, we have removed the corresponding CHA density panel from the supplementary figures. We greatly appreciate the reviewer's careful evaluation of our data presentation.

**Fig 2. Structural basis of G6P recognition by SLC37A4.**

(a) Superposition of the SLC37A4 apo state (grey) and the G6P-bound state (NTD in yellow, CTD in cyan). The structures are shown as cylindrical cartoons viewed along the membrane plane. The bound G6P molecule is shown as red sticks in the central cavity.

(b) View of the superposed apo and G6P‑bound structures from the ER‑lumen side along the membrane normal, highlighting G6P and nearby residues R28, K29, Y60, K64, F237 and K240.

(c) Cryo-EM density of the bound G6P molecule, with a contour level of 3 σ.

(d) View of the interface between the N‑terminal and C‑terminal domains from the cytosolic side along the membrane normal, showing gating residues at the domain interface, including D72, R126, T136, D285, T354 and T364.

(e) Electrostatic surface representation of SLC37A4 viewed from the same orientation in b

(f) Two‑dimensional schematic of the G6P‑binding sites, depicting hydrogen‑bond interactions between G6P and K64, as well as hydrophobic contacts with F237 and Y60.

(g) GFP-based thermostability assay showing G6P-induced changes in melting temperature (ΔT_m_) for wild type (WT) SLC37A4 and G6P-binding site mutants (R28A, R28C, R28H, K29A, Y60A, K64A, K240A). Bars represent mean ± s.d. from at least three technical replicates.
(h) Relative [^13^C]G6P uptake for WT and mutant SLC37A4. Bars represent mean ± s.d. from at least three technical replicates.

**Fig 3. Structural basis of inhibition of SLC37A4 by CHA**

(a) Overall structure of CHA‑bound SLC37A4 shown as a cylindrical cartoon in the cytosol-facing conformation. The N‑terminal domain NTD is in yellow and the C‑terminal domain CTD is in cyan. The bound inhibitor CHA is shown in magenta.

(b) View of the NTD–CTD interface from the ER‑lumen side along the membrane normal, highlighting polar and charged residues at the interdomain interface, including K46, D47, T157 in the NTD and S263, A388 and K389 in the CTD.

(c) Surface representation of SLC37A4 in the same orientation as in b, showing the interdomain interface patch formed by T157, K46 and D47 in the NTD and S263, A388 and K389 in the CTD.

(d) View of SLC37A4 from the cytosolic side along the membrane normal, showing the CHA was trapped at the interface of NTD and CTD.

(e) Close‑up view of the CHA‑binding pocket, highlighting residues that form hydrophobic interactions with CHA, including W118, F134, W138 and G135 from the NTD and N354, G350, V351, H366 and V369 from the CTD.

(f) Cryo-EM density of the bound CHA molecule, with a contour level of 8σ.

(g) Two‑dimensional schematic of the CHA‑binding site, illustrating hydrophobic contacts and van der Waals interactions between CHA and surrounding residues G135, F134, W138, W118, N354, G350, V351, H366, V369 and G370.

(h) Structural comparison of the apo (grey) and CHA‑bound (yellow/cyan) SLC37A4 structures, viewed from the membrane plane (left), from the cytosol (middle), and separated into NTD and CTD (right), illustrating that CHA binding induces a rigid‑body rotation between the two domains without major intra‑domain rearrangement.

3.) Thermal shift data

There is no information on the concentrations of the substrates used for this assay and overall the assay is described quite poorly. What has been done? How were the data analyzed? I recommend showing at least as a supplementary figure the thermal transitions curves for each mutant (+/- substrate) including the fit to a two state unfolding model. Comparing the thermal shift at a single concentration can often also be quite misleading, since the absolute shift is not always a measure of affinity. I recommend to measure a concentration dependent shift for the different mutants and determine apparent Kds. This allows a much better comparison of the mutants. A bit puzzling are the data on the K64A mutant - although identified as crucial in coordinating the substrate, the mutant obviously still binds almost as wild type. Do you have any idea or speculation as to why this is the case?

**Response:** We thank the reviewer for their detailed and constructive feedback regarding the thermal shift assay.

First, we have expanded the Methods section in the revised manuscript to fully describe the assay conditions, explicitly stating the substrate concentration used (10 mM G6P) and detailing the data analysis procedures. As requested, we have also included the full thermal transition curves for both the WT and mutant proteins. These are now provided as Supplementary Figure 5 (and are embedded below for your convenience).

Second, we agree with the reviewer that determining apparent Kds via concentration-dependent shifts would be ideal for comparing the mutants. However, our data suggest that the overall binding affinity of G6P to SLC37A4 is relatively weak. For context, during our structural optimization, we failed to resolve G6P density in a cryo-EM dataset collected in the presence of 30 mM G6P (data not included in the manuscript), and we ultimately required a concentration of 100 mM G6P to successfully resolve the ligand density presented in our final structure. Consistent with this weak apparent affinity, we found that lower substrate concentrations (e.g., 1 mM G6P) did not induce significant ΔT_m_ in the thermal shift assay. Because higher concentrations are required to observe a shift, the assay unfortunately did not yield the reliable concentration-dependent saturation curves needed for accurate Kd calculations. Therefore, we performed the assay at 10 mM G6P to ensure a measurable shift and have retained this single-concentration analysis, which provides a reliable qualitative assessment of substrate-induced stabilization.

Finally, we share the reviewer's puzzlement regarding the K64A mutant, which exhibits a wild-type-like thermal shift despite being identified as crucial. While the exact biochemical reason for this remains unclear, it suggests that K64 may be strictly essential for the conformational changes required during the transport cycle rather than initial substrate binding, or that the thermal shift assay is simply not sensitive enough to capture subtle binding defects for this specific residue. Importantly, our newly added transport activity assay (Figure 2h) clearly demonstrates that the K64A mutation significantly impairs transport function. This confirms its critical role in the overall transport cycle, even if its thermal stability profile in the presence of the substrate resembles the wild type. We have updated the manuscript text to reflect these findings and discuss this interesting observation. We thank the reviewer for helping us clarify this section.

**Supplementary Fig 5. Thermal transition curves of wild-type and mutant SLC37A4.**
(a–h) GFP-based thermal shift assays for wild-type (WT) SLC37A4 (a) and mutants R28A (b), R28C (c), R28H (d), K29A (e), Y60A (f), K64A (g), and K240A (h). The normalized fluorescence intensity (FI) signal is plotted as a function of temperature. Assays were performed in the absence (grey circles) or presence (red triangles) of 10 mM glucose-6-phosphate (G6P). Data were from at least triplicates. The calculated apparent melting temperatures (Tm) for each individual replicate are indicated in the corresponding panel legends.

4.) Comparison to recently published G6PT structures (PMID: 41136424 and 41225049) and related work.

The current manuscript lacks functional transport data to directly validate or challenge the structural interpretations. However, recent studies on SLC37A4 have already reported extensive biochemical and transport data (on substrates, inhibitors and mutational effects) that could be integrated and discussed in the context of the presented structures. This structure-function analysis would strengthen the manuscript.

The authors begin to compare their structures with those from recent publications (Supplementary Figure 5) and state in the Discussion that the results are largely similar. Nevertheless, closer inspection of the G6P-bound structures reveals notable differences that should be discussed in more detail. In particular, the authors should clarify which structural features are conserved across studies and where do they differ. A more systematic comparison addressing shared and distinct features across the available G6PT structures would help readers better understand how the present work extends, refines, or revises the existing structural framework for SLC37A4.

**Response:** We thank the reviewer for the excellent suggestion to compare our models with the recently published SLC37A4 structures (PMIDs: 41136424 and 41225049). We agree that a systematic comparison strengthens the manuscript, and we have significantly expanded our Discussion to address the shared and distinct features across these models.

Briefly, our comparison highlights the following key points:

- Conserved Architecture and Inhibitor Binding: Our models show remarkable convergence with the external datasets. The global architecture and the specific binding mode of the inhibitor CHA are virtually identical across all studies, providing powerful cross-validation of the overall structural framework (Supplementary Fig. 8).
- Distinct Substrate Coordination: In contrast to the inhibitor, the precise coordination of G6P varies considerably among the models. While the position of the H366 side chain remains largely unchanged across all structures, its involvement in substrate binding differs dramatically. In our structure, the phosphate group interacts with Y60 and K64, but the glucose moiety points away, leaving H366 at a distance of 10.7 Å with no direct interaction. Conversely, in PDBs 9W1P and 9KVV, the substrate forms close contacts (2.5 Å to 3.3 Å) with H366 (Supplementary Fig. 9c).
- Mechanistic Interpretation: Because G6P in our structure lacks this additional coordination from H366, it exhibits a looser binding mode involving fewer overall protein contacts. We interpret this as a distinct intermediate state along the transport cycle. It likely captures a transient moment of initial substrate engagement or imminent release, trapped by subtle differences in sample preparation or lipid environments.

By detailing these shared and distinct structural features in the revised text, we hope to provide readers with a clearer understanding of how our work complements and extends the existing structural framework for SLC37A4.Minor points:

Supplementary Figures 2-4 C: should read 'model'

Supplementary Figures 2-4 D: unit Å is missing

**Response**: We thank the reviewer for catching these omissions. We have corrected the labels in Supplementary Figures 2, 3, and 6 (formerly Supplementary Figure 4). Specifically, panel C now correctly reads 'model', and the missing 'Å' unit has been added to panel D in all three figures.
