# Supplementary material for: Structural basis for substrate recognition and inhibition of human glucose-6-phosphate transporter SLC37A4
Source: PLoS Biol. 2026 Jul 28;24(7):e3003833. doi: 10.1371/journal.pbio.3003833 (PMC13411879; doi:10.1371/journal.pbio.3003833)
Supplement: Peer review history

# Editor report: Decision Letter 0

**DOI:** 10.1371/journal.pbio.3003833.r001

**Authors:** Richard Hodge

6 Jan 2026

Dear Dr Wei,

Thank you for submitting your manuscript entitled "Structural insight into substrate recognition and inhibition of human glucose 6-phosphate transporter SLC37A4" for consideration as a Short Report by PLOS Biology. Please accept my sincere apologies for the delay in getting back to you due to the closure of the editorial office during the recent holiday period.

Your manuscript has now been evaluated by the PLOS Biology editorial staff, as well as by an academic editor with relevant expertise, and I am writing to let you know that we would like to send your submission out for external peer review under our complementary research policy.

However, before we can send your manuscript to reviewers, we need you to complete your submission by providing the metadata that is required for full assessment. To this end, please login to Editorial Manager where you will find the paper in the 'Submissions Needing Revisions' folder on your homepage. Please click 'Revise Submission' from the Action Links and complete all additional questions in the submission questionnaire.

Once your full submission is complete, your paper will undergo a series of checks in preparation for peer review. After your manuscript has passed the checks it will be sent out for review. To provide the metadata for your submission, please Login to Editorial Manager (https://www.editorialmanager.com/pbiology) within two working days, i.e. by Jan 08 2026 11:59PM.

If your manuscript has been previously peer-reviewed at another journal, PLOS Biology is willing to work with those reviews in order to avoid re-starting the process. Submission of the previous reviews is entirely optional and our ability to use them effectively will depend on the willingness of the previous journal to confirm the content of the reports and share the reviewer identities. Please note that we reserve the right to invite additional reviewers if we consider that additional/independent reviewers are needed, although we aim to avoid this as far as possible. In our experience, working with previous reviews does save time.

If you would like us to consider previous reviewer reports, please edit your cover letter to let us know and include the name of the journal where the work was previously considered and the manuscript ID it was given. In addition, please upload a response to the reviews as a 'Prior Peer Review' file type, which should include the reports in full and a point-by-point reply detailing how you have or plan to address the reviewers' concerns.

During the process of completing your manuscript submission, you will be invited to opt-in to posting your pre-review manuscript as a bioRxiv preprint. Visit http://journals.plos.org/plosbiology/s/preprints for full details. If you consent to posting your current manuscript as a preprint, please upload a single Preprint PDF.

Feel free to email us at plosbiology@plos.org if you have any queries relating to your submission.

Kind regards,

Richard

Richard Hodge, PhD

Senior Editor, PLOS Biology

rhodge@plos.org

PLOS

Empowering researchers to transform science

Carlyle House, Carlyle Road, Cambridge, CB4 3DN, United Kingdom

California (U.S.) corporation #C2354500, based in San Francisco

# Peer review: Decision Letter 1

**DOI:** 10.1371/journal.pbio.3003833.r002

**Authors:** Richard Hodge

4 Feb 2026

Dear Dr Wei,

Thank you for your patience while your manuscript "Structural insight into substrate recognition and inhibition of human glucose 6-phosphate transporter SLC37A4" was peer-reviewed at PLOS Biology. Please accept my apologies for the delays that you have experienced during the peer review process. Your manuscript has now been evaluated by the PLOS Biology editors, an Academic Editor with relevant expertise, and by two independent reviewers.

In light of the reviews, which you will find at the end of this email, we would like to invite you to revise the work to thoroughly address the reviewers' reports.

As you will see below, the reviewers think the glucose-6-phosphate transporter structure is interesting and of high-quality, but they raise overlapping concerns with the lack of mutational validation and further mechanistic insight in the manuscript. Specifically, Reviewer #1 notes that transport assays should be provided to strengthen the functional relevance of the work and Reviewer #2 asks that thermal shift assay data for each mutant are provided, as well as providing a comparison with recently published G6PT structures.

Given the extent of revision needed, we cannot make a decision about publication until we have seen the revised manuscript and your response to the reviewers' comments. Your revised manuscript is likely to be sent for further evaluation by all or a subset of the reviewers.

In addition to these revisions, you will need to complete some formatting changes, which you will receive in a follow up email. A member of our team will be in touch with a set of requests shortly.

We expect to receive your revised manuscript within 3 months. Please email us (plosbiology@plos.org) if you have any questions or concerns, or would like to request an extension.

At this stage, your manuscript remains formally under active consideration at our journal; please notify us by email if you do not intend to submit a revision so that we may withdraw it.

**IMPORTANT - SUBMITTING YOUR REVISION**

Your revisions should address the specific points made by each reviewer. Please submit the following files along with your revised manuscript:

1. A 'Response to Reviewers' file - this should detail your responses to the editorial requests, present a point-by-point response to all of the reviewers' comments, and indicate the changes made to the manuscript.

*NOTE: In your point-by-point response to the reviewers, please provide the full context of each review. Do not selectively quote paragraphs or sentences to reply to. The entire set of reviewer comments should be present in full and each specific point should be responded to individually, point by point.

You should also cite any additional relevant literature that has been published since the original submission and mention any additional citations in your response.

2. In addition to a clean copy of the manuscript, please also upload a 'track-changes' version of your manuscript that specifies the edits made. This should be uploaded as a "Revised Article with Changes Highlighted" file type.

*Re-submission Checklist*

When you are ready to resubmit your revised manuscript, please refer to this re-submission checklist: https://plos.io/Biology_Checklist

To submit a revised version of your manuscript, please go to https://www.editorialmanager.com/pbiology/ and log in as an Author. Click the link labelled 'Submissions Needing Revision' where you will find your submission record.

Please make sure to read the following important policies and guidelines while preparing your revision:

*Published Peer Review*

Please note while forming your response, if your article is accepted, you may have the opportunity to make the peer review history publicly available. The record will include editor decision letters (with reviews) and your responses to reviewer comments. If eligible, we will contact you to opt in or out. Please see here for more details:

https://blogs.plos.org/plos/2019/05/plos-journals-now-open-for-published-peer-review/

*PLOS Data Policy*

Please note that as a condition of publication PLOS' data policy (http://journals.plos.org/plosbiology/s/data-availability) requires that you make available all data used to draw the conclusions arrived at in your manuscript. If you have not already done so, you must include any data used in your manuscript either in appropriate repositories, within the body of the manuscript, or as supporting information (N.B. this includes any numerical values that were used to generate graphs, histograms etc.). For an example see here: http://www.plosbiology.org/article/info%3Adoi%2F10.1371%2Fjournal.pbio.1001908#s5

*Blot and Gel Data Policy*

We require the original, uncropped and minimally adjusted images supporting all blot and gel results reported in an article's figures or Supporting Information files. We will require these files before a manuscript can be accepted so please prepare them now, if you have not already uploaded them. Please carefully read our guidelines for how to prepare and upload this data: https://journals.plos.org/plosbiology/s/figures#loc-blot-and-gel-reporting-requirements

*Protocols deposition*

To enhance the reproducibility of your results, we recommend that if applicable you deposit your laboratory protocols in protocols.io, where a protocol can be assigned its own identifier (DOI) such that it can be cited independently in the future. Additionally, PLOS ONE offers an option for publishing peer-reviewed Lab Protocol articles, which describe protocols hosted on protocols.io. Read more information on sharing protocols at https://plos.org/protocols?utm_medium=editorial-email&utm_source=authorletters&utm_campaign=protocols

Thank you again for your submission to our journal. We hope that our editorial process has been constructive thus far, and we welcome your feedback at any time. Please don't hesitate to contact us if you have any questions or comments.

Best regards,

Richard

Richard Hodge, PhD

Senior Editor, PLOS Biology

rhodge@plos.org

------------------------------------

REVIEWS:

Reviewer #1: In this work, the authors report the cryo-EM structures of human SLC37A4 in apo, G6P-bound, and CHA-bound states, providing an important structural framework for substrate recognition and inhibition. The manuscript is well written. Although a few highly related studies have been published recently, considering the scope of PLOS Biology, this work remains valuable and should be suitable for publication if the following concerns are properly addressed.

1. Transport assay

As SLC37A4 is a G6P/Pi exchanger, the mechanistic conclusions would be substantially strengthened by adding a transport assay (e.g., using reconstituted proteoliposomes or a cellular transport assay). Some mutational analysis using a transport assay should be performed in the revision.

2. Terminology for CHA as "allosteric"

The authors describe CHA as binding to an "allosteric" site. However, the structures show that the CHA-interacting region partially overlaps with the G6P-binding region. Therefore, it is unclear whether it is appropriate to refer to this site as allosteric. Thus, if the authors continue to use the term "allosteric," please provide a more detailed explanation and justification. Otherwise, please refrain from using "allosteric".

3 Typos

Page 17, Line 16 (Data Availability)

"SCL37A4" should be corrected as "SLC37A4".

"EMD-64869" should be corrected as "EMD-67869".

Supplementary Figure 5.

In panel d, "Y64" should be corrected as "Y60".

In panel e, "PDB 9KVO" should be corrected as "PDB 9KV0".

Reviewer #2: In this manuscript, Li et al. report cryo-EM structures of the human glucose-6-phosphate transporter (G6PT/SLC37A4) in the apo and G6P-bound states, as well as in complex with the allosteric inhibitor chlorogenic acid (CHA). These structures capture the transporter in both inward- and outward-facing conformations characteristic of Major Facilitator Superfamily transporters, allowing them to draw conclusions about the conformational changes occurring during the transport cycle. Overall, the study provides new insights into the structure, function, and conformational dynamics of the SLC37 family. The structural work is of high quality, but I have several comments that may help to further strengthen the manuscript.

1.) Modeling of the cytoplasmic loop connecting the N- and C-bundle

The authors did not specify which portion of the cytoplasmic loop connecting TM6 and TM7 was modeled. A clear description and labeling in the figures would be helpful. Are there any disease related mutations located?

2.) Comments to figures

- all figures (main plus supplementary) illustrating EM densities require a statement on the shown contour level

- label termini (at least in the first figure); loops or parts not modelled should be shown in dashed lines

- enlarge the density for G6P in figure 2 and show from at least two different orientations

- highlight EM density for CHA in figure 3 (not only in supplementary figure)

3.) Thermal shift data

There is no information on the concentrations of the substrates used for this assay and overall the assay is described quite poorly. What has been done? How were the data analyzed? I recommend showing at least as a supplementary figure the thermal transitions curves for each mutant (+/- substrate) including the fit to a two state unfolding model. Comparing the thermal shift at a single concentration can often also be quite misleading, since the absolute shift is not always a measure of affinity. I recommend to measure a concentration dependent shift for the different mutants and determine apparent Kds. This allows a much better comparison of the mutants. A bit puzzling are the data on the K64A mutant - although identified as crucial in coordinating the substrate, the mutant obviously still binds almost as wild type. Do you have any idea or speculation as to why this is the case?

4.) Comparison to recently published G6PT structures (PMID: 41136424 and 41225049) and related work.

The current manuscript lacks functional transport data to directly validate or challenge the structural interpretations. However, recent studies on SLC37A4 have already reported extensive biochemical and transport data (on substrates, inhibitors and mutational effects) that could be integrated and discussed in the context of the presented structures. This structure-function analysis would strengthen the manuscript.

The authors begin to compare their structures with those from recent publications (Supplementary Figure 5) and state in the Discussion that the results are largely similar. Nevertheless, closer inspection of the G6P-bound structures reveals notable differences that should be discussed in more detail. In particular, the authors should clarify which structural features are conserved across studies and where do they differ. A more systematic comparison addressing shared and distinct features across the available G6PT structures would help readers better understand how the present work extends, refines, or revises the existing structural framework for SLC37A4.

Minor points:

Supplementary Figures 2-4 C: should read 'model'

Supplementary Figures 2-4 D: unit Å is missing

# Author comment: Author response to Decision Letter 2

**DOI:** 10.1371/journal.pbio.3003833.r003

13 Apr 2026

Attachment Submitted filename: response-041026.docx

# Peer review: Decision Letter 2

**DOI:** 10.1371/journal.pbio.3003833.r004

**Authors:** Richard Hodge

13 May 2026

Dear Dr Wei,

Thank you for your patience while we considered your revised manuscript entitled "Structural insight into substrate recognition and inhibition of human glucose 6-phosphate transporter SLC37A4" for publication as a Short Report at PLOS Biology. This revised version of your manuscript has been evaluated by the PLOS Biology editors, the Academic Editor and the two original reviewers.

Based on the reviews (attached below), we are likely to accept this manuscript for publication provided you document better the cellular transport studies in the methods, as requested by the Academic Editor, and that you reduce the main figures to a total of four to comply with the requirements of our Short Reports. Please also make sure to address the policy-related requests stated below my signature.

In addition, we would like you to consider a suggestion to improve the title:

“Structural basis for substrate recognition and inhibition of the human glucose 6-phosphate transporter SLC37A4”

As you address these items, please take this last chance to review your reference list to ensure that it is complete and correct. If you have cited papers that have been retracted, please include the rationale for doing so in the manuscript text, or remove these references and replace them with relevant current references. Any changes to the reference list should be mentioned in the cover letter that accompanies your revised manuscript.

In addition to these revisions, you may need to complete some formatting changes, which you will receive in a follow up email. A member of our team will be in touch with a set of requests shortly. If you do not receive a separate email within a few days, please assume that checks have been completed, and no additional changes are required.

We expect to receive your revised manuscript within two weeks.

To submit your revision, please go to https://www.editorialmanager.com/pbiology/ and log in as an Author. Click the link labelled 'Submissions Needing Revision' to find your submission record. Your revised submission must include the following:

- a cover letter that should detail your responses to any editorial requests, if applicable, and whether changes have been made to the reference list

- a Response to Reviewers file that provides a detailed response to the reviewers' comments (if applicable, if not applicable please do not delete your existing 'Response to Reviewers' file.)

- a track-changes file indicating any changes that you have made to the manuscript.

NOTE: If Supporting Information files are included with your article, note that these are not copyedited and will be published as they are submitted. Please ensure that these files are legible and of high quality (at least 300 dpi) in an easily accessible file format. For this reason, please be aware that any references listed in an SI file will not be indexed. For more information, see our Supporting Information guidelines:

https://journals.plos.org/plosbiology/s/supporting-information

*Published Peer Review History*

Please note that you may have the opportunity to make the peer review history publicly available. The record will include editor decision letters (with reviews) and your responses to reviewer comments. If eligible, we will contact you to opt in or out. Please see here for more details:

https://plos.org/published-peer-review-history/

*Press*

Should you, your institution's press office or the journal office choose to press release your paper, please ensure you have opted out of Early Article Posting on the submission form. We ask that you notify us as soon as possible if you or your institution is planning to press release the article.

*Protocols deposition*

To enhance the reproducibility of your results, we recommend that if applicable you deposit your laboratory protocols in protocols.io, where a protocol can be assigned its own identifier (DOI) such that it can be cited independently in the future. Additionally, PLOS ONE offers an option for publishing peer-reviewed Lab Protocol articles, which describe protocols hosted on protocols.io. Read more information on sharing protocols at https://plos.org/protocols?utm_medium=editorial-email&utm_source=authorletters&utm_campaign=protocols

Please do not hesitate to contact me should you have any questions.

Sincerely,

Ines

--

Ines Alvarez-Garcia, PhD

Senior Editor

PLOS Biology

ialvarez-garcia@plos.org

on behalf of

Richard Hodge, PhD

Senior Editor

PLOS Biology

rhodge@plos.org

------------------------------------------------------------------------

DATA POLICY:

You may be aware of the PLOS Data Policy, which requires that all data be made available without restriction: http://journals.plos.org/plosbiology/s/data-availability. For more information, please also see this editorial: http://dx.doi.org/10.1371/journal.pbio.1001797

Note that we do not require all raw data. Rather, we ask that all individual quantitative observations that underlie the data summarized in the figures and results of your paper be made available in one of the following forms:

1) Supplementary files (e.g., excel). Please ensure that all data files are uploaded as 'Supporting Information' and are invariably referred to (in the manuscript, figure legends, and the Description field when uploading your files) using the following format verbatim: S1 Data, S2 Data, etc. Multiple panels of a single or even several figures can be included as multiple sheets in one excel file that is saved using exactly the following convention: S1_Data.xlsx (using an underscore).

2) Deposition in a publicly available repository. Please also provide the accession code or a reviewer link so that we may view your data before publication.

Regardless of the method selected, please ensure that you provide the individual numerical values that underlie the summary data displayed in the following figure panels as they are essential for readers to assess your analysis and to reproduce it:

Fig. 2G-H and Fig. S5A-H

NOTE: the numerical data provided should include all replicates AND the way in which the plotted mean and errors were derived (it should not present only the mean/average values).

Please also ensure that figure legends in your manuscript include information on WHERE THE UNDERLYING DATA CAN BE FOUND, and ensure your supplemental data file/s has a legend.

Please ensure that your Data Statement in the submission system accurately describes where your data can be found.

**In addition, please make the structures publicly available at this stage.

------------------------------------------------------------------------

CODE POLICY

Per journal policy, if you have generated any custom code during the course of this investigation, please make it available without restrictions. Please ensure that the code is sufficiently well documented and reusable, and that your Data Statement in the Editorial Manager submission system accurately describes where your code can be found. More information on our Code Policy, what and how to share can be found here: https://journals.plos.org/plosbiology/s/code-availability

Please note that we cannot accept sole deposition of code in GitHub, as this could be changed after publication. However, you can archive this version of your publicly available GitHub code to Zenodo. Once you do this, it will generate a DOI number, which you will need to provide in the Data Accessibility Statement (you are welcome to also provide the GitHub access information). See the process for doing this here: https://docs.github.com/en/repositories/archiving-a-github-repository/referencing-and-citing-content

-------------------------------------------------------------------------

BLOT AND GEL REPORTING REQUIREMENTS:

We require the original, uncropped and minimally adjusted images supporting all blot and gel results reported in an article's figures or Supporting Information files. We will require these files before a manuscript can be accepted so please prepare and upload them now. Please carefully read our guidelines for how to prepare and upload this data: https://journals.plos.org/plosbiology/s/figures#loc-blot-and-gel-reporting-requirements

------------------------------------------------------------------------

Reviewers' comments

Reviewer #1:

The authors addressed all of my concerns.

Reviewer #2:

The authors have substantially revised the manuscript and have addressed all of my previous comments and concerns. Although the revisions required a considerable amount of work, I feel the effort was worthwhile. The implemented functional data and the comparison with the previously published structures nicely complement this work. I recommend the manuscript for publication.

# Editor report: Decision Letter 3

**DOI:** 10.1371/journal.pbio.3003833.r005

**Authors:** Richard Hodge

18 May 2026

Dear Dr Wei,

On behalf of my colleagues and the Academic Editor, Raimund Dutzler, I am pleased to say that we can accept your manuscript for publication, provided you address any remaining formatting and reporting issues. These will be detailed in an email you should receive within 2-3 business days from our colleagues in the journal operations team; no action is required from you until then. Please note that we will not be able to formally accept your manuscript and schedule it for publication until you have completed any requested changes.

Please take a minute to log into Editorial Manager at http://www.editorialmanager.com/pbiology/, click the "Update My Information" link at the top of the page, and update your user information to ensure an efficient production process.

PRESS

We frequently collaborate with press offices. If your institution or institutions have a press office, please notify them about your upcoming paper at this point, to enable them to help maximise its impact. If the press office is planning to promote your findings, we would be grateful if they could coordinate with biologypress@plos.org. If you have previously opted in to the early version process, we ask that you notify us immediately of any press plans so that we may opt out on your behalf.

We also ask that you take this opportunity to read our Embargo Policy regarding the discussion, promotion and media coverage of work that is yet to be published by PLOS. As your manuscript is not yet published, it is bound by the conditions of our Embargo Policy. Please be aware that this policy is in place both to ensure that any press coverage of your article is fully substantiated and to provide a direct link between such coverage and the published work. For full details of our Embargo Policy, please visit http://www.plos.org/about/media-inquiries/embargo-policy/.

Thank you again for choosing PLOS Biology for publication and supporting Open Access publishing. We look forward to publishing your study.

Best wishes,

Richard

Richard Hodge, PhD

Senior Editor, PLOS Biology

rhodge@plos.org

PLOS

Empowering researchers to transform science

Carlyle House, Carlyle Road, Cambridge, CB4 3DN, United Kingdom

California (U.S.) corporation #C2354500, based in San Francisco
